# Development of a Transformation System for *Chlamydia trachomatis*: Restoration of Glycogen Biosynthesis by Acquisition of a Plasmid Shuttle Vector

**DOI:** 10.1371/journal.ppat.1002258

**Published:** 2011-09-22

**Authors:** Yibing Wang, Simona Kahane, Lesley T. Cutcliffe, Rachel J. Skilton, Paul R. Lambden, Ian N. Clarke

**Affiliations:** 1 Molecular Microbiology Group, University of Southampton Medical School, Southampton General Hospital, Southampton, United Kingdom; 2 Department of Virology, Faculty of Health Sciences, Ben Gurion University of the Negev, Beer Sheva, Israel; Duke University, United States of America

## Abstract

*Chlamydia trachomatis* remains one of the few major human pathogens for which there is no transformation system. *C. trachomatis* has a unique obligate intracellular developmental cycle. The extracellular infectious elementary body (EB) is an infectious, electron-dense structure that, following host cell infection, differentiates into a non-infectious replicative form known as a reticulate body (RB). Host cells infected by *C. trachomatis* that are treated with penicillin are not lysed because this antibiotic prevents the maturation of RBs into EBs. Instead the RBs fail to divide although DNA replication continues. We have exploited these observations to develop a transformation protocol based on expression of β-lactamase that utilizes rescue from the penicillin-induced phenotype. We constructed a vector which carries both the chlamydial endogenous plasmid and an *E.coli* plasmid origin of replication so that it can shuttle between these two bacterial recipients. The vector, when introduced into *C. trachomatis* L2 under selection conditions, cures the endogenous chlamydial plasmid. We have shown that foreign promoters operate *in vivo* in *C. trachomatis* and that active β-lactamase and chloramphenicol acetyl transferase are expressed. To demonstrate the technology we have isolated chlamydial transformants that express the green fluorescent protein (GFP). As proof of principle, we have shown that manipulation of chlamydial biochemistry is possible by transformation of a plasmid-free *C. trachomatis* recipient strain. The acquisition of the plasmid restores the ability of the plasmid-free *C. trachomatis* to synthesise and accumulate glycogen within inclusions. These findings pave the way for a comprehensive genetic study on chlamydial gene function that has hitherto not been possible. Application of this technology avoids the use of therapeutic antibiotics and therefore the procedures do not require high level containment and will allow the analysis of genome function by complementation.

## Author Summary

*C. trachomatis* is a major human pathogen for which there is no means of genetically manipulating its DNA. It is an obligate intracellular bacterium which has a complex developmental cycle that takes place in a specialized host cell cytoplasmic vacuole known as an inclusion. We have constructed a shuttle vector based on the chlamydial plasmid and developed a new approach to select genetically modified bacteria. It uses rescue by selection of stable infectious, penicillin-resistant *C. trachomatis* from a pool of non-dividing, non-infectious *C. trachomatis* (induced by penicillin arrest of the developmental cycle). The transformed *C. trachomatis* is also cured of its endogenous plasmid by the selection of the transforming vector. The vector was modified to express the green fluorescent protein (GFP) in both *Chlamydia* and *E. coli*. We have genetically manipulated a plasmid-free recipient strain of *C. trachomatis* and shown restoration of the ability of this recipient strain to synthesize and accumulate glycogen within inclusions upon acquisition of the shuttle vector. The ability to transform and manipulate *C. trachomatis* using a complementation based vector is an important advance that opens up the field of *Chlamydia* research.

## Introduction

*C. trachomatis* is a major human pathogen with a unique intracellular developmental cycle [1], [2]. This cycle begins when the extracellular, infectious form of the microorganism, the EB, binds to susceptible host cells [3], [4]. EBs are taken up into a phagocytic vesicle which is modified to become a chlamydial inclusion where individual EBs differentiate into the metabolically active, replicative form of the microorganism, the RB [5]. RBs divide by binary fission and, when 8–10 divisions have elapsed, they differentiate back into EBs that are released by cell lysis [6].

*C. trachomatis* has a small and highly conserved genome of some 1,000 kb [7]. In addition, most *C. trachomatis* isolates carry a plasmid of 7.5kb [8] which encodes eight genes. All eight genes are transcribed [9] and translated during the developmental cycle [10]. Despite the availability of the plasmid as a potential vector, the development of a simple robust genetic transformation system for the *chlamydiae* has remained a significant challenge [11]. The demand for such a system is evidenced by the recent development of a means to mutate the *C. trachomatis* chromosome [12] but to reach its full potential this requires a complementary gene transfer system. An approach using chromosomal integration was used to make recombinants in *C. psittaci* EBs by allelic exchange using exogenous DNA introduced by electroporation [13]. This was limited to the 16S rDNA region and only allowed integration of a short 1 kb marker at extremely low efficiencies. *C. psittaci* requires high levels of containment and therefore is not readily available to the wider research community. Electroporation of EBs was used in 1994 in an attempt to transform *C. trachomatis* with an episomal vector based on the chlamydial plasmid [14]. No stable transformants were isolated although inclusions were present for up to four passages under chloramphenicol selection. There have been reports of plasmid free strains of *C. trachomatis* but only three viable, naturally occurring plasmid-free isolates of *C. trachomatis* have been described [15]–[17], thus most *C. trachomatis* isolates carry the 7.5 kbp plasmid, its biological function is unknown although its presence has for a long time been linked to the ability of *C. trachomatis* to synthesise glycogen [18]. We tried unsuccessfully to cure a lymphogranuloma venereum (LGV) strain of *C. trachomatis* L2 of its plasmid [19] but recently curing of the plasmid from a genital tract *C. trachomatis* D has been described [20]. This plasmid-cured strain and the naturally occurring plasmid-free strains do not stain for glycogen [21]; the plasmid does not encode glycogen synthesis genes thus the phenomenon involves complex interaction(s) between the plasmid and the chlamydial genome to elicit the glycogen staining phenotype [22]. However, formal proof associating this property with the plasmid alone is necessary but can only be achieved by re-introducing the plasmid into a plasmid-free strain. This has not been possible because of the absence of a means to genetically manipulate *C. trachomatis*.

We report here the first successful development of a simple, robust, reproducible plasmid-based genetic transformation system for *C. trachomatis* using penicillin selection and calcium chloride (CaCl_2_) treatment of EBs to render them competent. Penicillin causes *C. trachomatis* to enter a persistent non-infectious state [6], [23]–[25]; therefore our experimental plan was to select genetically stable, penicillin-resistant transformants by recovery of *Chlamydiae* from penicillin-arrested division. To demonstrate the effectiveness and reproducibility of the procedure, we have engineered a strain of *C. trachomatis* that is penicillin resistant and that expresses GFP. We have also proven the role of the chlamydial plasmid in glycogen biosynthesis by re-introducing the plasmid into a *C. trachomatis* strain that is plasmid-free (*C. trachomatis* L2 (25667R)) [15]. We have demonstrated that as a result of this genetic transformation the previously plasmid-free strain *C. trachomatis* L2 (25667R) acquired the ability to synthesize and accumulate glycogen within inclusions.

## Results/Discussion

Our primary aim was to develop a transformation protocol using a chlamydial plasmid-based shuttle vector. Our scientific aim was to determine whether glycogen biosynthesis, a distinctive characteristic of *C. trachomatis* which has been linked to the presence of the plasmid, could be restored in a plasmid-free, glycogen-free variant of *C. trachomatis*, by re-introducing the plasmid DNA.

### Plasmid shuttle vector - design and choice of selectable marker(s)

**Figure 1:**
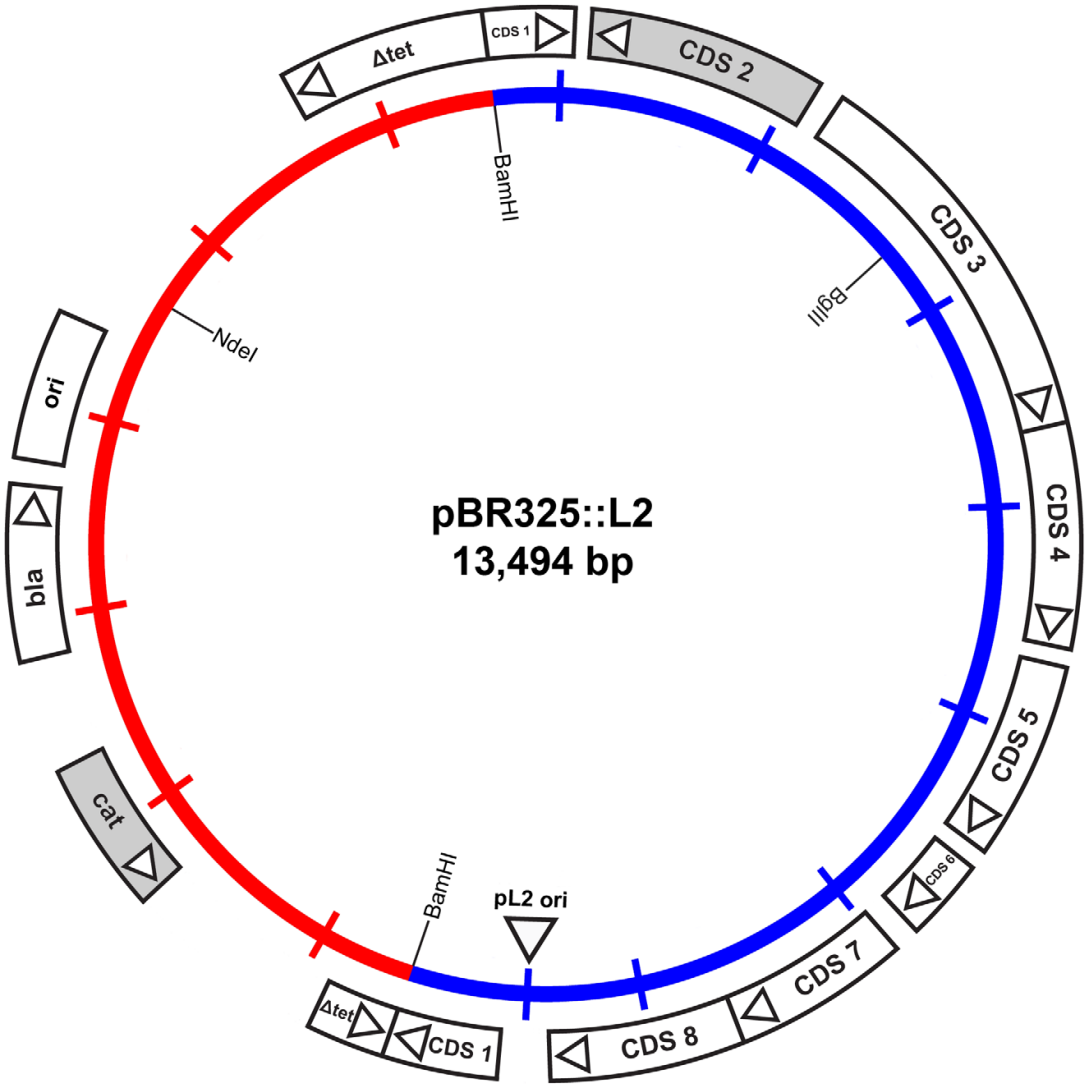
Map of the shuttle vector pBR325::L2. The inner circle represents the plasmid pL2 from *C. trachomatis* L2/434/Bu (blue) and the vector sequences of pBR325 (red). The coding sequences and their direction of transcription are represented by the boxes of the outer circle. The β-lactamase (*bla*), chloramphenicol acetyl transferase (*cat*) and inactivated tetracycline resistance gene (*Δtet*) are as indicated, as are key restriction endonuclease cleavage sites.

To enable the selection of transformants for plasmid vectors, antibiotic resistance markers offer the most attractive choices. There are a range of single gene antibiotic resistance markers that are safely used worldwide for the routine and stable transformation of bacteria. These markers are popular because it is not possible to generate transformants unless an intact gene is acquired thus eliminating the problem of background resistance through spontaneous mutation. The markers used in routine selection of bacterial transformants include tetracycline resistance, chloramphenicol resistance and β-lactamase (penicillin/ampicillin resistance) [26]. Tetracycline resistance markers have been used in allelic transfer experiments for chlamydiae [27], but tetracycline is a controversial choice because it is used routinely to treat *C. trachomatis* infections [28]. *C. trachomatis* is sensitive to chloramphenicol but it is difficult to reproduce a minimal inhibitory assay as this antibiotic causes mitochondrial stress [29] limiting chlamydial growth and is thus not so useful for selection and continuous passaging of transformants. By contrast, the effects of penicillin on *C. trachomatis* are well studied [6], [25], it is bacteriostatic giving a resistant phenotype and penicillin is not recommended for the treatment of chlamydial infections [30]. Following penicillin treatment, the developmental cycle is slowed and the transition to EBs ceases with the formation of giant, aberrant RBs. The normal developmental cycle resumes upon removal of penicillin (at low concentrations) from the culture media and the resultant normal chlamydial inclusions are easily detectable by phase contrast microscopy [6]. We reasoned that rescue of infectious *C. trachomatis* from the penicillin-induced aberrant developmental cycle through transformation and β-lactamase expression would present a distinctive phenotype that could be easily selected microscopically and transformants could be recovered under penicillin selection. We tested a range of penicillin concentrations on the growth of normal *C. trachomatis* L2 and determined that 10 units/ml of penicillin was a suitable concentration for inhibition of *C. trachomatis* and hence this was the antibiotic concentration chosen for the *C. trachomatis* transformation work. Penicillin resistance was introduced into a *C. trachomatis* plasmid (pL2) by ligating a pBR325 plasmid and the pL2 plasmid (pBR325::L2). This was a simple, standard recombinant plasmid from our collection that had an intact β-lactamase gene and the complete chlamydial plasmid. Ligation into the *Bam* HI site of pBR325 plasmid created an insertional inactivation of the *tet* gene but still conferred penicillin and chloramphenicol resistance. The *Bam* HI site in the pL2 plasmid is located in coding sequence 1 (CDS1), a region which is susceptible to mutation/deletion without affecting plasmid stability [8], [19]. The plasmid map is shown in Figure 1.

### Strains of C. trachomatis

We chose the well-studied, laboratory-adapted strain *C. trachomatis* L2/434/Bu (ATCC VR902B) as the recipient strain because we already had a full complement of biological and genetic information for this strain (the complete genome sequence and defined proteome) [31], [32]. The vector with which we started the work contained the cognate pL2 plasmid cloned from *C. trachomatis* L2/434/Bu genomic DNA [33]. Furthermore, the LGV strains [34] that we used: *C. trachomatis* L2/434/Bu and (later) the plasmid-free *C. trachomatis* L2 (25667R) have relatively low particle to infectivity ratios [35] and do not need centrifugation to achieve efficient cell infection, thus these bacteria have a higher viability than standard genital tract isolates and have a faster developmental cycle giving a quicker turn around of experiments [4].

### Transformation of *C. trachomatis* L2/434/Bu

We wanted to investigate whether it was possible to transform EBs using a simple standard protocol and a defined buffer that could be reproduced in any laboratory. Standard bacterial transformations are based on the primary observation that bacteria treated with ice cold solutions of CaCl_2_ followed by brief heating can be induced to take up foreign DNA [36]. Therefore we initially used such a protocol, developed for *E. coli*, to attempt transformation of gradient purified *C. trachomatis* L2/434/Bu EBs and selected transformants with 10 units/ml penicillin. However, we found that heat shock was not necessary and that the whole transformation protocol could be achieved at room temperature (RT) simply by gently mixing EBs, vector DNA and McCoy cells in CaCl_2_/Tris buffer. This protocol is described in the Materials and Methods and the selection procedure is described in Table S1. For selection our reasoning was to allow recovery of transformants, without penicillin selection in the first round of infection (developmental cycle) and then apply selection with penicillin initially at 10 units/ml (10 units  = 6 µg penicillin). After three rounds of selection, all the untransformed penicillin-inhibited *C. trachomatis* were lost and the culture was overgrown by penicillin-resistant transformants that appeared to grow with similar kinetics and inclusion morphology as the parental strain. It was possible to increase the concentration of penicillin to 20 and 100 units/ml at passages 3 and 4 to speed up the selection process. In each experiment regular observation of the cultures under phase contrast microscopy allows some flexibility in deciding precisely when the next passage is needed, taking into account the size, form and number of inclusion bodies observed at each stage.

The frequency of transformation, defined as the proportion of EBs or trypsinised cells receiving the shuttle vector DNA and still keeping the ability to start chlamydial proliferation, is a parameter that cannot be measured. The efficiency of recovery is dependent on the percentage of the transformed EBs present in the total population of EBs after each stage of selection. Each passage is performed by infecting cell cultures with cell lysates from the previous passage (see Materials and Methods, and Table S1). Following lysis of the McCoy cells, the untransformed *Chlamydia,* present as non-infectious RBs, fail to passage. Thus only transformed EBs and a diminishing number of carry-over untransformed EBs infect new host cells. The recovery increases with each passage until only transformed *Chlamydia* remain.

**Figure 2:**
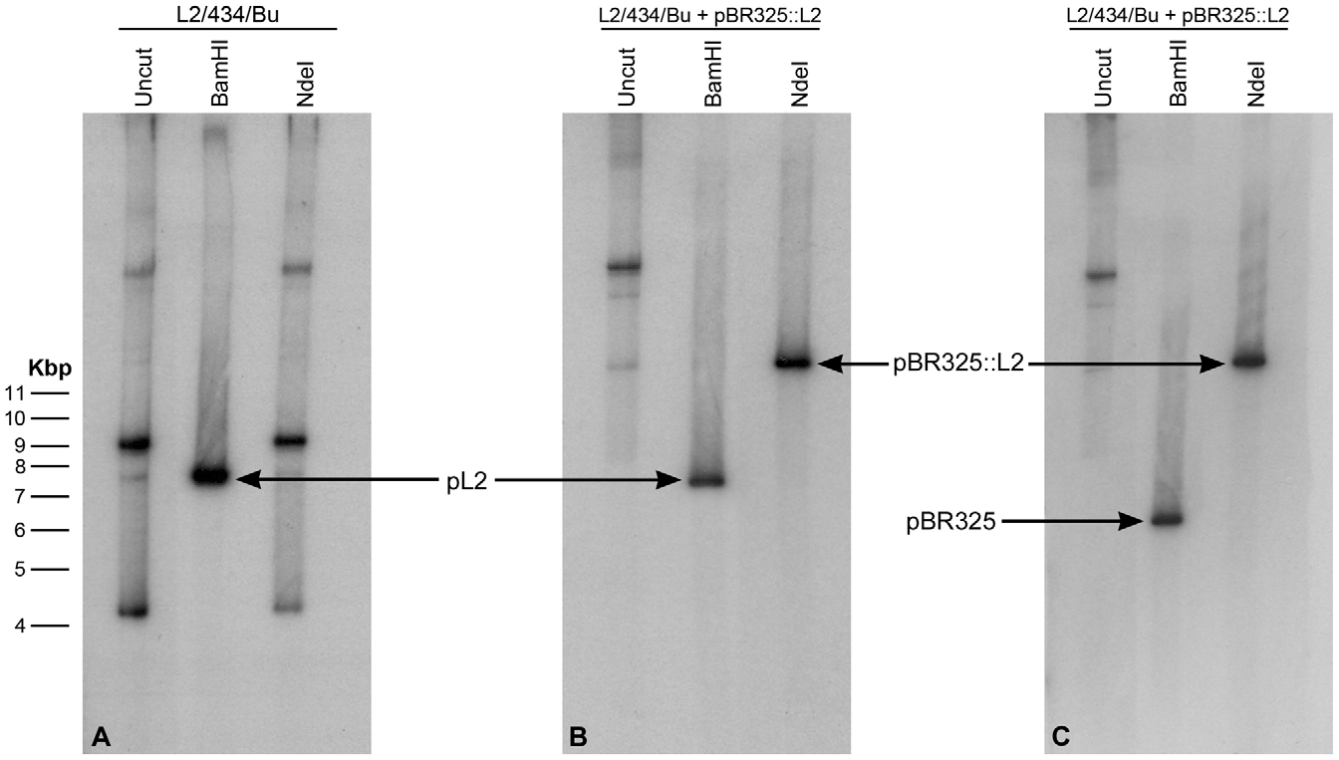
Southern blot of *C. trachomatis* L2/434/Bu transformed by plasmid pBR325::L2. Equal amounts of *C. trachomatis* DNA were loaded in each gel track, the DNA is uncut (track 1) or has been digested to completion with *Bam* HI (track 2) or *Nde* I (track 3). (A) DNA from wild type (untransformed *C. trachomatis* L2/434/Bu) was probed with the complete plasmid sequence pL2. (B) DNA from the transformed strain also probed with pL2. (C) DNA from the transformed strain probed with the complete β-lactamase gene sequence (generated by PCR). The wild type pL2 plasmid (7.5 kb) carries a unique *Bam* HI restriction site (which is used as the cloning site for insertion into the vector pBR325) but no *Nde* I site. The bands in tracks 1 and 3 in panel A represent various forms of the plasmid pL2. The recombinant plasmid pBR325::L2 is ∼13.5 kb, thus digestion with *Bam* HI releases the pBR325 vector (6 Kb – hybridizing to the *bla* probe in (C) and the chlamydial plasmid pL2 (7.5 kb – hybridizing to pL2 probe in panel B). The recombinant plasmid (pBR325::L2) carries a unique *Nde* I site (located in pBR325) and digestion with this enzyme linearises the vector at 13.5 kb; this band is detected with both hybridization probes (track 3 panels B and C).

The pBR325::L2 transformed *C. trachomatis* L2/434/Bu strain was plaque purified (x3), expanded, and EBs and RBs were purified for detailed characterization. Southern blotting of chromosomal DNA purified from EBs with a vector probe (β-lactamase gene) and a chlamydial plasmid probe proved that transformation had occurred and revealed no changes in either the *E. coli* vector (pBR325) or the L2 plasmid (pL2), although by these passages the endogenous chlamydial plasmid had been eliminated from the transformed strain (Figure 2). There was no evidence for recombination/integration of the transforming plasmid with the recipient *C. trachomatis* chromosomal DNA.

### Properties of pBR325::L2 transformed *C. trachomatis* L2/434/Bu

The copy number of pBR325::L2 was similar to that of the endogenous pL2 as accurately measured by qPCR (Figure S1).

**Figure 3:**
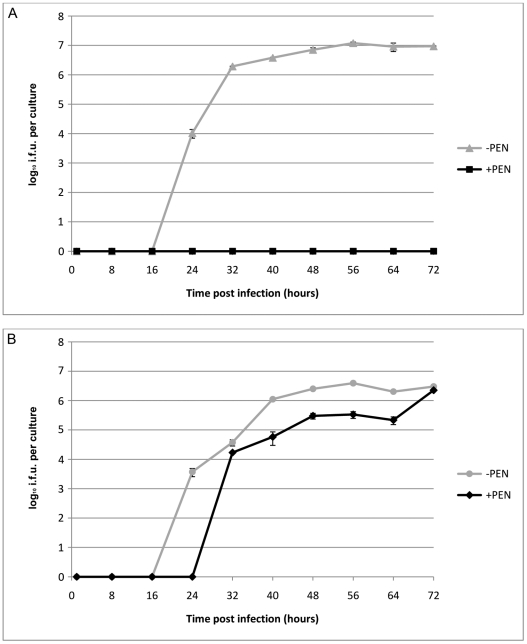
Growth characteristics of *C. trachomatis* L2 and *C. trachomatis* L2 transformed by pBR325::L2. (A) *C. trachomatis* L2/434/Bu, (B) *C. trachomatis* L2/434/Bu transformed by shuttle vector pBR325::L2. McCoy cells in a 24 well tissue culture tray grown to confluence were infected with *C. trachomatis* at MOI  = 1 and were cultured without (-PEN) and with (+PEN) penicillin G at 10 units/ml. The yield of *C. trachomatis* (IFU) per culture or single well is shown on the y – axis and time of sampling post infection is shown on the x –axis. The experiments were repeated in quadruplicate and standard error bars are shown for each sample point.

To investigate the effects of transformation by plasmid pBR325::L2 on the strain *C. trachomatis* L2/434/Bu, we compared the growth characteristics of the parental strain and the transformed strain with or without penicillin selection. These data are summarized in Figure 3 and clearly show that the parental strain (L2) grows well in McCoy cells, as previously demonstrated, and there was no recovery of infectious EBs in the presence of penicillin when it was added at 10 units/ml from the start of the developmental cycle.

By contrast, the transformed strain grew in both the presence (10 units/ml) and absence of penicillin (up to 8 passages) giving similar recoveries of infectious EBs at the end of developmental cycle. Nevertheless the presence of a large transforming plasmid (pBR325::L2) has a measurable effect and the developmental cycle was lengthened and the yield slightly reduced compared to the untransformed *C. trachomatis* L2/434/Bu. Transmission electron microscopy of infected cells at late stages of the developmental cycles show no obvious phenotypic differences for transformed or untransformed *C. trachomatis* in the absence of penicillin but in the presence of penicillin untransformed *C. trachomatis* produced large aberrant RBs whereas transformed *C. trachomatis* inclusions appeared normal (Figure S2).

The vector was recovered from pBR325::L2- transformed *C. trachomatis* L2 by genomic DNA preparation and analysed by direct sequencing. These data showed no evidence for the presence of the original pL2 and also confirmed that there were no rearrangements or recombination events with pL2 or within the pBR325::L2 vector, although there were a few base changes from the sequences deposited in the database for the original pBR325 vector (Figure S3). These were attributable to errors in the original sequencing and annotation rather than adaptive mutations or subsequent transformation of the DNA in to *E. coli*. Analysis of multiple colonies carrying the recovered pBR325::L2 from *E. coli* by mini-plasmid preparation and restriction digestion showed they were all clonal and appeared to be unchanged from the transforming vector. One of these recovered plasmids was also sequenced and was identical to the sequence obtained direct from the transformed *C. trachomatis.*

Immunoblotting of purified EBs and RBs with commercially available antibodies (Figure S4) showed the presence of both β-lactamase and chloramphenicol acetyl transferase enzymes consistent with the observation that pBR325::L2- transformed *C. trachomatis* L2 was, in contrast to the untransformed *C. trachomatis* L2, resistant to penicillin up to 100 units/ml and able to grow in medium containing chloramphenicol concentrations up to 3 µg/ml (data not shown).

Interestingly, both the processed and mature forms of β-lactamase were detectable by immunoblotting in EBs showing that the signal peptide [37] was functional in *C. trachomatis* and the pre-protein was cleaved completely in RBs (Figure S4A). Chloramphenicol acetyl transferase levels (as assessed by immunoblot) were the same in both RBs and EBs (Figure S4B).

**Figure 4:**
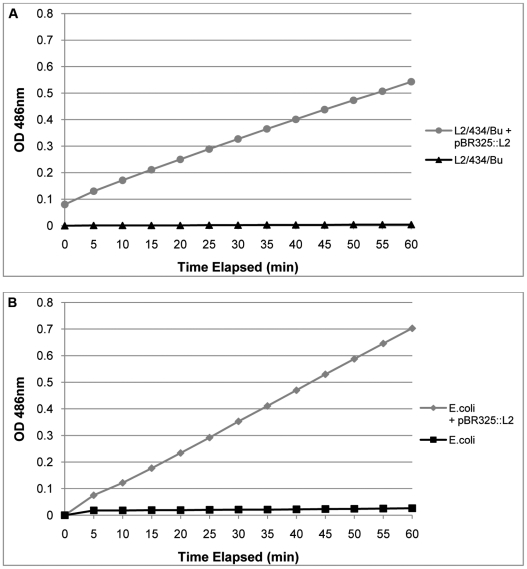
Purified, transformed RBs have β – lactamase activity. In Panel A equal amounts of purified RBs (measured by qPCR of the genome) from transformed *C. trachomatis* L2/434/Bu and the wild–type parental *C. trachomatis* L2/434/Bu were assayed for β – lactamase activity. In Panel B equal amounts of *E. coli* and *E. coli* transformed with pBR325::L2 were used as controls to verify the assay. Hydrolysis of Nitrocefin is measured in pH 7.0 buffer by absorbance at 486 nm over 60 min. Nitrocefin is a chromogenic β – lactamase substrate.

The transformed *C. trachomatis* L2 grew under penicillin selection and whilst the presence of β-lactamase, as shown by Western blotting, indicated that resistance to penicillin is likely through the mechanism of action of this enzyme, it did not prove that the enzyme is active. Thus to prove formally that the resistance to penicillin was indeed due to acquisition of an active β-lactamase, purified RBs (the actively growing form of *C. trachomatis*) were assayed for β-lactamase activity. Figure 4 shows that pBR325::L2- transformed *C. trachomatis* RBs have β-lactamase activity whereas RBs from the untransformed parental strain do not.

These results, taken together, established that pBR325::L2 could be selected from a background of untransformed *C. trachomatis* and this vector could also replicate in *E. coli*, therefore providing the basis of a functional shuttle vector. They showed proof of principle that the functions for retaining and replicating the plasmid in *C. trachomatis* were unaffected in the shuttle vector and we have established that standard *E. coli* promoters for both β-lactamase and chloramphenicol acetyl transferase operate in *C. trachomatis*. Further, the β-lactamase is active and the type 1 signal peptidase mechanism from *C. trachomatis* [38] is able to cleave the β-lactamase precursor to a mature form of the correct molecular weight in RBs, the actively growing form of the micro-organism.

In our experiments deriving a transformation frequency as a percentage of EBs receiving vector DNA is not a relevant measure and we have no means of measuring how many survive selection in the first round of culture. We considered it might be possible to do this if we had a marker such as green fluorescence that we could use to identify live transformed *Chlamydia*.

### Expression of the green fluorescent protein in *C. trachomatis* L2/434/Bu

**Figure 5:**
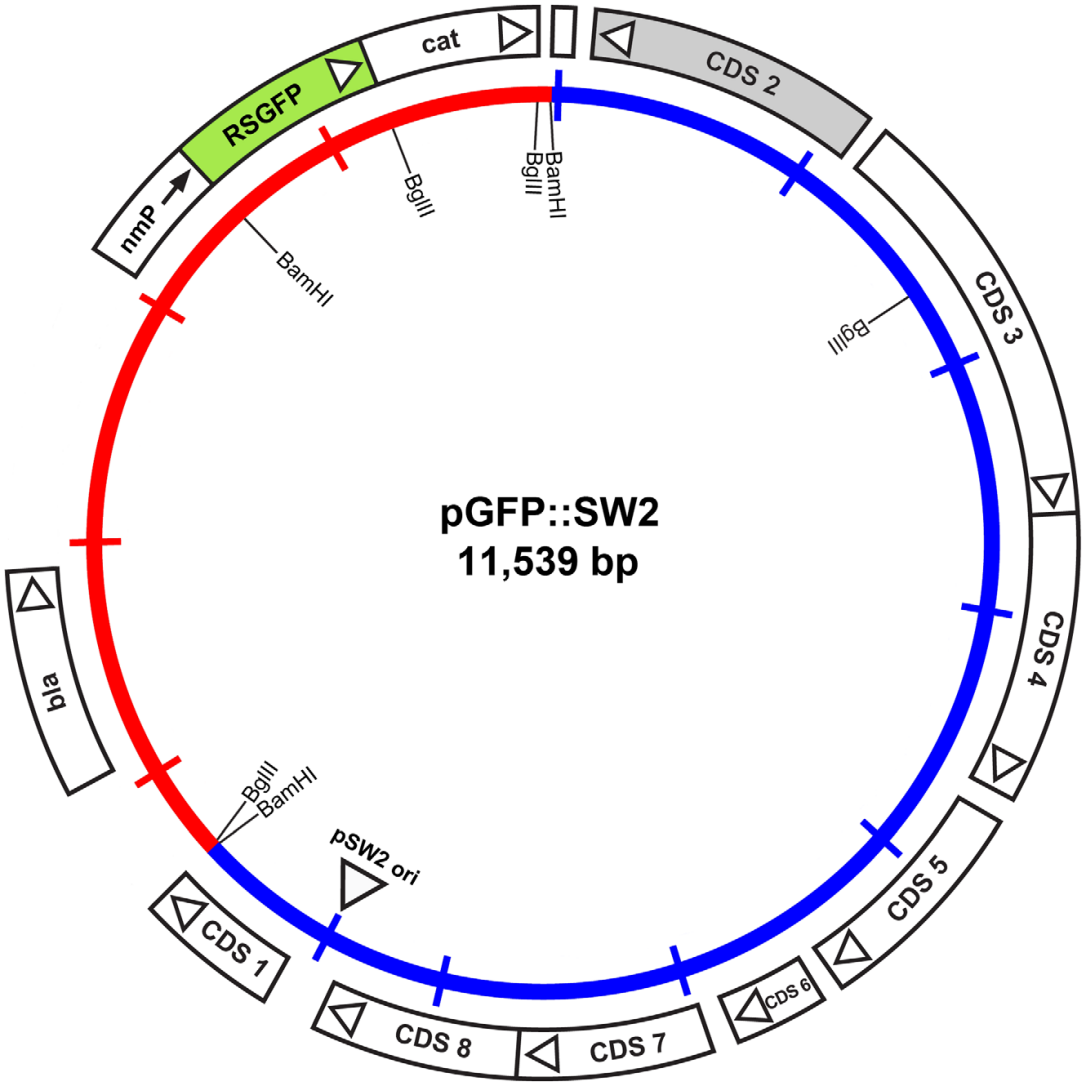
Map of the plasmid vector pGFP::SW2. The inner circle represents the plasmid pSW2 from *C. trachomatis* SW2 (blue) and the vector sequences (red). The coding sequences and their direction of transcription are represented by the boxes of the outer circle. Some key restriction endonuclease cleavage sites used in the construction of this vector are indicated on the inner circle. The *cat* gene is fused with RSGFP (shaded green) and expressed by a promoter derived from *Neisseria meningitidis* (nmP); the direction of transcription of this promoter is designated by the arrow.

To show that the transformants obtained in the first series of experiments were useful for general application and not just the outcome from a serendipitous choice of a single, vector configuration (pBR325::L2) it was essential to repeat the work but with more than one (and different) marker. For this purpose we chose the mutated plasmid from the Swedish new variant which has a characteristic 377 bp deletion in CDS1 and a 44 bp duplication in the 5′ terminus of CDS3 [8]. This plasmid was cloned into an *E. coli* vector able to express the GFP. The complete plasmid map of the shuttle vector carrying the mutated plasmid from SW2 is shown in Figure 5.

Plasmid pGFP::SW2 contains a β-lactamase gene together with a red-shifted green fluorescent protein gene fused to the chloramphenicol acetyl transferase gene under control of a neisserial promoter. The map of the original plasmid pRSGFPCAT that provided the fused *rsgfp-cat* cassette including the neisserial promoter is shown in Figure S5.

*E. coli* cells transformed with pRSGFPCAT are resistant to penicillin and chloramphenicol and fluoresce green under blue illumination (data not shown).

The plasmids and DNA used to make this final shuttle vector (pGFP::SW2) are summarized in Figure S6. The complete sequence and list of features for pGFP::SW2 are summarized in Figure S7.

**Figure 6:**
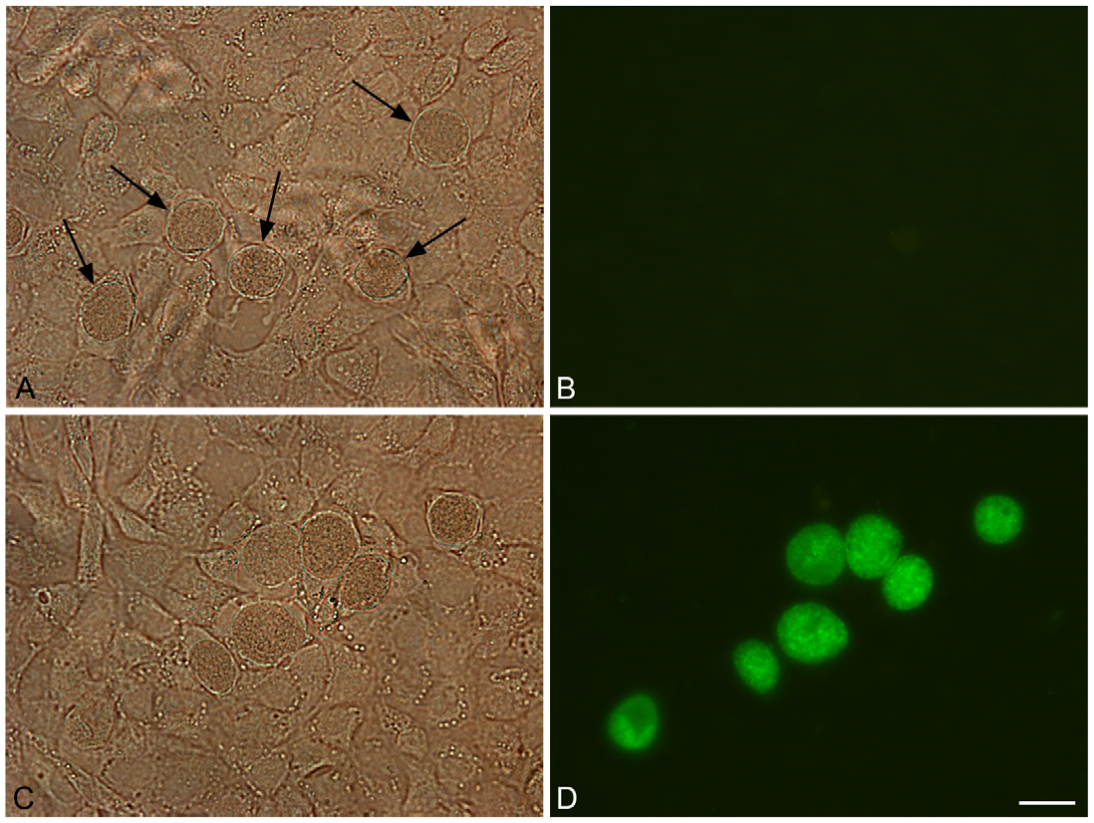
Green fluorescent inclusions in McCoy cells infected with *C. trachomatis* L2/434/Bu transformed with pGFP::SW2. Untransformed *C. trachomatis* L2/434/Bu (control) and *C. trachomatis* L2/434/Bu transformed by pGFP::SW2 were grown on coverslips for two days before fixing in 4% formaldehyde (diluted in DPBS). Panel A shows untransformed *C. trachomatis* L2/434/Bu under white light (arrows indicate inclusions). Panel B is the same image under blue light. Panel C shows *C. trachomatis* L2/434/Bu transformed with plasmid pGFP::SW2 under white light, and panel D is the same field under blue light. The scale bar represents 20 µm.

Transformation of *C. trachomatis* L2/434/Bu with pGFP::SW2 was successfully achieved on six occasions and yielded a penicillin-resistant strain that had green fluorescent inclusions (Figure 6). Green fluorescent inclusions became visible at 24 h post infection, once the transformants had been selected by multiple passages; however, we were unable to observe green fluorescent inclusions in the first developmental cycle post-transformation.

The green fluorescent protein is expressed as a fusion protein with the chloramphencol acetyl transferase (GFPCAT). To show that the fluorescence observed in inclusions was derived from the GFPCAT fusion protein, transformed *Chlamydia* were immunoblotted with anti-GFP monoclonal antibodies as shown in Figure S8.

### Transformation of plasmid-free *C. trachomatis* with pGFP::SW2 restores the ability to biosynthesise glycogen

**Figure 7:**
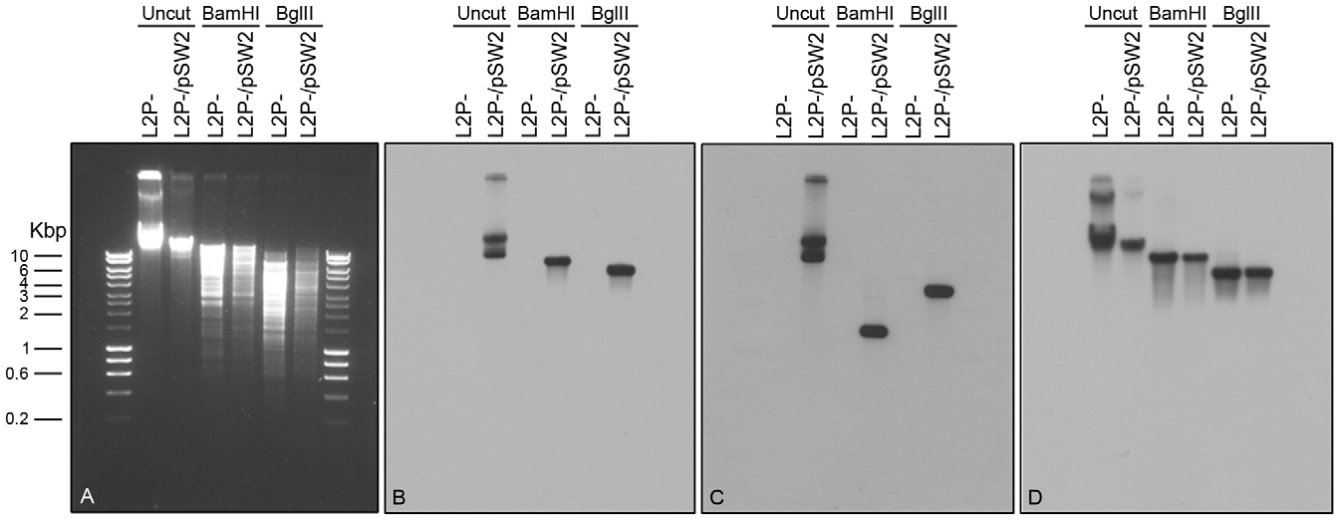
Southern blot of *C. trachomatis* L2 (25667R) transformed by plasmid pGFP::SW2. Southern hybridization analyses of plasmid-free *C. trachomatis* L2 (25667R) (abbreviated as L2P-) and *C. trachomatis* L2 (25667R) transformed with plasmid pGFP::SW2 (in short L2P-/pSW2). Six chlamydial genomic DNA samples were loaded on the gel in pairs together with HyperLadder I (5 µl) from BIOLINE (Cat No. BIO-33025). The agarose gel image was taken before DNA transfer (A). The DNA blot was first hybridized with the pSW2 probe (B), and then stripped and re-probed with the GFP probe (C) or an *ompA* probe (D). *Bam* HI digestion of pGFP::SW2 created 3 fragments: 7169 bp (containing pSW2 probe sequence), 2925 bp and 1445 bp (containing GFP probe sequence). *Bgl* II digestion of pGFP::SW2 created 4 fragments: 5555 bp (containing the pSW2 probe sequence), 3625 bp (containing the GFP probe sequence), 1693 bp and 666 bp. When the *C. trachomatis* L2 genomic DNA (similar to L2P-) was digested with *Bam* HI or *Bgl* II, the fragment containing *ompA* sequence was 8837 bp (*Bam* HI digestion) or 5518 bp (*Bgl* II digestion). Southern hybridization using the pSW2 probe or the GFP probe showed hybridization signals at expected positions in all L2P-/pGFP::SW2 samples (uncut or digested); whilst no hybridization signal, as expected, was detected in all L2P- samples (uncut or digested) (B) and (C). Southern hybridization using the *ompA* probe showed that the hybridization signals were similar in L2P- and L2P-/pGFP::SW2 samples (uncut or digested) (D).

The ability to stain for the presence of glycogen in chlamydial inclusions is a property that has been linked to the presence of the plasmid although it has not been possible to prove formally the association as no system has previously existed for the introduction of plasmids into *C. trachomatis* [21]. The *C. trachomatis* L2 (25667R) strain is plasmid-free and not able to accumulate glycogen. We were unsure whether it would be possible to transform this strain as it was plasmid-free and may have lost the ability to retain the plasmid. Nevertheless, we attempted transformation of this *C. trachomatis* L2 strain with the vector pGFP::SW2 and obtained stable penicillin-resistant transformants that had green fluorescent inclusions. However, transformation of *C. trachomatis* L2 (25667R) strain with the basic cloning plasmid pSP73 alone did not yield transformants showing that the chlamydial plasmid (pSW2) was necessary to provide the replication functions of the shuttle vector pGFP::SW2 in *C. trachomatis* [8], [19]. We were able to recover the intact pGFP::SW2 vector from transformed *C. trachomatis* L2 (25667R) by genomic DNA preparation and re-transform the DNA into *E. coli*. Southern blotting of DNA extracted from transformed plasmid-free *C. trachomatis* L2 (25667R) confirmed the presence of pGFP::SW2 (Figure 7) and proved that transformation had occurred.

**Figure 8:**
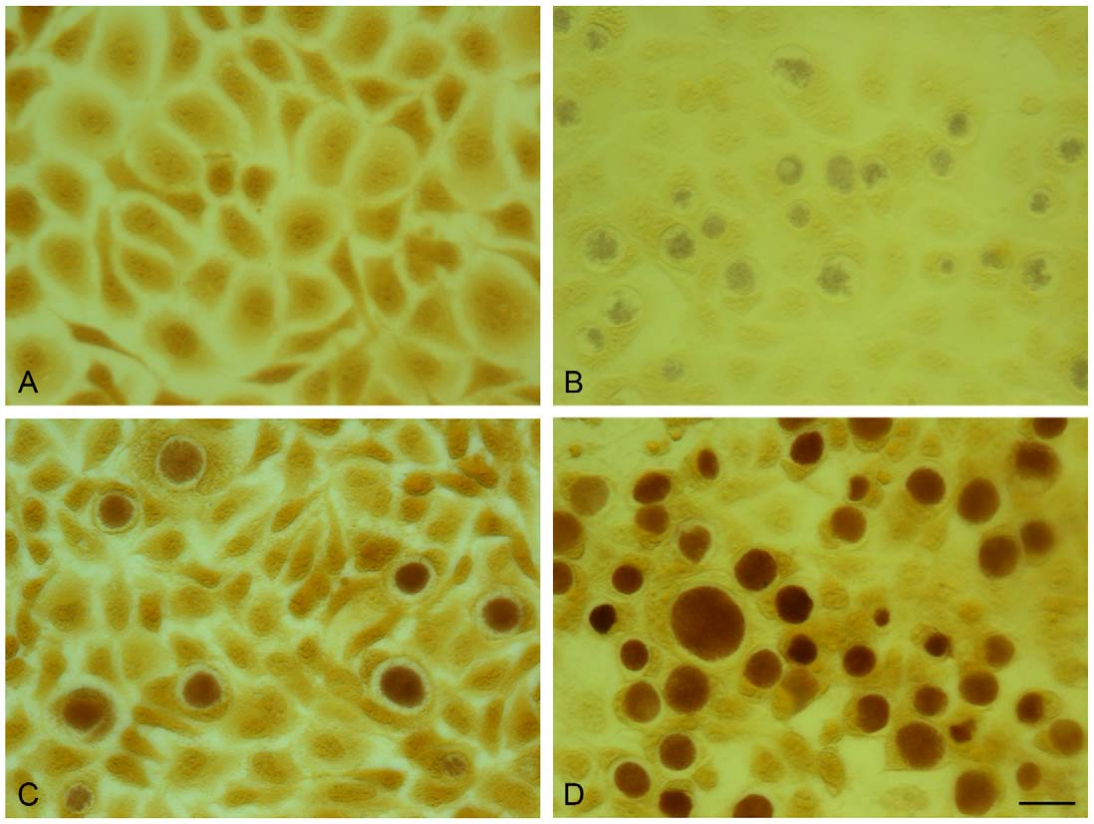
Acquisition of the plasmid pGFP::SW2 restores the ability of plasmid-free *C. trachomatis* L2 (25667R) to synthesize glycogen. The presence of glycogen within inclusions was detected by iodine staining of cells on coverslips. (A) McCoy cells control, (B) *C. trachomatis* L2 (25667R) in McCoy cells, (C) *C. trachomatis* L2/434/Bu in McCoy cells, (D) pGFP::SW2- transformed *C. trachomatis* L2 (25667R) in McCoy cells. The scale bar represents 20 µm.

The pGFP::SW2-transformed *C. trachomatis* L2 (25667R) had inclusions which stained positive for glycogen (Figure 8). This observation confirms that glycogen biosynthesis is a trait that is dependent on the presence of the chlamydial plasmid. Every inclusion from the pGFP::SW2-transformed *C. trachomatis* L2 (25667R) stained for glycogen, demonstrating stable transformation at the individual level as well as the population level (as shown by the Southern blots).

### Conclusions

1. We have developed a simple and reproducible genetic transformation protocol for *C. trachomatis* based on calcium chloride treatment of EBs.
2. We have designed a shuttle vector based on the 7.5 kb chlamydial plasmid and the *E. coli* plasmid pBR325. This vector replicates in both *C. trachomatis* and *E. coli* and selection is based on penicillin resistance.
3. *C. trachomatis* L2/434/Bu transformed by the shuttle vector pBR325::L2 shows similar growth characteristics and inclusion formation as the untransformed strain.
4. We have developed a second smaller shuttle vector pGFP::SW2 that conveys penicillin resistance through beta-lactamase expression and that also expresses the GFP.
5. *C. trachomatis* L2/434/Bu transformed with pGFP::SW2 produces green fluorescent inclusions.
6. Transformation of a plasmid-free strain of *C. trachomatis* L2 (25667R) with pGFP::SW2 generates green fluorescent inclusions and also restores the glycogen staining phenotype confirming that this property is a plasmid-dependent property.
7. The development of a simple and reproducible transformation method will open the way to a better understanding of chlamydial genetics and could lead to the development of new approaches to chlamydial vaccines and therapeutic interventions aimed at modulating chlamydial pathogenesis.

## Materials and Methods

### Microscopy

Cells in culture and cells infected with *C. trachomatis* L2 were routinely visualized by phase contrast microscopy using a Nikon eclipse TS100 inverted microscope with fluorescence accessories. Fluorescence images were captured using a Leica DMRB microscope to visualize the expression of GFP in McCoy cells infected by pGFP::SW2-transformed *C. trachomatis* L2. Counting of inclusion forming units (IFU) to quantify chlamydial infectivity was performed on serial dilutions of *C. trachomatis* L2 in monolayers of McCoy cells grown in 96 well trays. For this assay inclusions were immunostained as previously described for *C. abortus* [39]. For transmission EM studies McCoy cells infected with *C. trachomatis* L2 at a multiplicity of infection (MOI) of 1 were grown in 6 well trays and 48 h post infection were fixed with 3% glutaraldehyde in 0.1% cacodylate buffer, processed as previously described [40] and photographed using an Hitachi H7000 electron microscope.

### *E. coli* strains, recombinant plasmids and genetic manipulations

*E. coli* strain DH5α [41], [42] and its derivative strain *E. coli* ‘Top10’ from Invitrogen were used for the basic cloning and construction of vectors pRSGFPCAT and pGFP::SW2. The vector pBR325::L2 was constructed by ligation of pL2 cleaved by *Bam* HI from plasmid PDCPB [33] (this vector is equivalent to pBR322::L2) into *Bam* HI cleaved pBR325 (GenBank: L08855.1), this cloning was performed in *E. coli* strain HB101. The original preparation of the plasmid pBR325::L2 DNA (used to transform *C. trachomatis* L2 EBs) was performed in *E. coli* strain HB101, but all subsequent plasmid manipulations for transformation were performed using *E. coli* GM 2163. This strain is mutated for *Dam*, *Dcm* and *Mcr* methylation systems and was available from New England Biolabs (Cat. no. #E4105S). Plasmid pGFP::SW2 (Figure 5) was constructed from the *C. trachomatis* SW2 plasmid pSW2, pSP73 and pRSGFPCAT. The complete cloning strategy is summarised in Figure S6. Plasmid pRSGFPCAT is a small in-house vector based on a pUC origin of replication and carrying the *cat* gene fused to RSGFP and under the control of a neisserial promoter that is constitutively expressed in *E. coli* (refer to Figure S5 for sequence details). Briefly, the *Bam* HI fragment of pSW2 (GenBank: FM865439.1 - obtained by gel extraction from a *C. trachomatis* SW2 total genomic DNA *Bam* HI digestion) (Figure S6 panel A) was cloned into the unique *Bam* HI site of plasmid pSP73 (Figure S6 panel B) to give plasmid pSP73::SW2 (Figure S6 panel C). The *Pst* I/*Sal* I fragment of pRSGFPCAT (Figure S6 panel D) was then cloned into *Pst* I/*Sal* I backbone of pSP73 allowing selection of ampicillin resistant, chloramphenicol resistant green fluorescent colonies.

### Ethics statement

All genetic manipulations and containment work was approved under the UK Health and Safety Executive Genetically Modified Organisms (contained use) regulations 2000 notification no GM57,10.1 entitled ‘Genetic transformation of Chlamydiae’.

### Cell culture and propagation of *C. trachomatis* strains

McCoy cells were used for propagation of *C. trachomatis* and for the transformation studies. Two strains of *C. trachomatis* were used as recipient strains for transformation in this study: *C. trachomatis* L2/434/Bu (ATCC VR-902B) which carries a 7.5 kb plasmid (pL2) and *C. trachomatis* L2 (25667R) which has no plasmid [15]. Both strains were confirmed as pure, clonal isolates by 3 rounds of plaque purification and, together with the McCoy cells, were regularly tested for mycoplasma contamination by fluorescence microscopy using Hoechst no. 33258 staining and by VenorGem Mycoplasma PCR detection (Minerva Biolabs, Berlin, Germany) according to the manufacturer's instructions. The McCoy cells were grown in Dulbecco's modified Eagles' medium (DMEM) supplemented with 10% fetal calf serum (FCS). Cell concentrations were determined by staining with Trypan Blue and counting in a haemocytometer. Cells were infected with *C. trachomatis* by overlay of the inoculum for 1 h in medium containing cycloheximide (1 µg/ml) and gentamicin (25 µg/ml). On completion of the developmental cycle and immediately prior to host cell lysis, infected monolayers were detached with trypsin/EDTA buffer (from Invitrogen, Cat. # 25300-054) and EBs were harvested in DMEM containing 10% FCS at 3,000 g for 10 min. The *C. trachomatis*-infected cell pellet was suspended in a solution of 10% PBS in water and homogenized in a Dounce homogenizer to break open the cells and release the EBs. Cell debris was removed by centrifugation at 250 g for 5 min and the supernatant containing partially purified EBs was mixed with an equal volume of phosphate/sucrose buffer (16 mM Na_2_HPO_4_ pH 7.1 and 0.4 M sucrose, abbreviated as 4SP), and was stored at −80°C.

### Plaque assay for infectivity assay and for clonal purification of EBs and RBs

The *C. trachomatis* strains were plaque purified as previously described [31], [43]. Briefly a single plaque was picked, cultured and purified to clonality by two further rounds of plaquing. The plaque-purified *Chlamydia* were then used to make stock preparations. Large scale cultures were prepared for the production of EBs and RBs which were purified by two cycles of Urografin (Schering Healthcare, UK) density gradient centrifugation as previously described [31].

### Genetic transformation of C. *trachomatis*

A simple protocol was developed where *C. trachomatis* L2 EBs were first mixed with plasmid DNA and then used to infect freshly trypsinised McCoy cells (MOI  = 2.5). The preparation of EBs, vector DNA and cells followed by the transformation protocol is described in detail below.

1. Preparation of EBs for transformation: McCoy cells were grown in 6× T_75_ flasks, and infected with *C. trachomatis* L2 in fresh medium (DMEM + 10%FCS) containing 1 µg/ml of cycloheximide, and grown in 37°C, 5% CO_2_ incubator for 2 days at an MOI  = 2 giving ∼90% cells infected. The cells were bulk harvested using cell scrapers, and then spun at 3500 rpm for 10 min. The cell pellet was saved, resuspended in 1 ml of cold 10% PBS, and transferred into a bijoux tube with glass beads. The cells were then lysed by vortexing for 1 min. The cell debris was removed by spinning at 1000 rpm for 5 min. The supernatant was saved (∼1 ml) and mixed with 1 ml of 4SP. The inocula were divided into 100 µl aliquots and stored at −80°C.

2. The vector DNA was extracted from overnight *E. coli* cultures (GM2163 strain) using the PureYield Plasmid Midiprep System (Promega Cat. No. A2492). The quality and the concentration of the DNA were evaluated by agarose gel electrophoresis and NanoDrop 1000 Spectrophotometer (from Thermo Scientific). The DNA was used at concentration ∼0.5–1 µg/µl.

3. McCoy cells were prepared for transformation when cells were ∼70% confluent in a T_75_ flask, the medium was removed and the cells were washed twice with 5 ml Dulbecco's PBS (DPBS) (from Invitrogen, Cat. # 14190-094). Then 2 ml Trypsin/EDTA buffer was added to cover the cells. Trypsinisation was allowed to proceed at RT for 5 min and when the cells were released from the flask, 10 ml of medium was added with a pipette, and the medium was washed up and down with the pipette to release the remaining cells and to break up clumps. The cells were transferred to a plastic universal and spun in a bench top centrifuge (Beckman Coulter Allegra X-15R) at 1000 rpm for 5 min, the medium was removed and the pellet briefly rinsed and then resuspended in 5 ml DPBS and pelleted again at 1000 rpm for 5 min. The DPBS buffer was discarded and cells resuspended in CaCl_2_ buffer for transformation.

4. Transformation is performed as follows: 10 µl *Chlamydia* EBs (1×10^7^ IFU) and 10 µl plasmid DNA (6 µg) were mixed in a total volume of 200 µl CaCl_2_ buffer (10 mM Tris pH 7.4 and 50 mM CaCl_2_) and then incubated for 30 min at room temperature. Freshly trypsinised McCoy cells (4×10^6^), resuspended in 200 µl CaCl_2_ buffer were then added to the plasmid/EB mix and incubated for a further 20 min at room temperature with occasional mixing. 100 µl of this mixture was then added to a single well in a six well tray together with 2 ml of pre-warmed DMEM + 10% FCS. The cells were allowed to settle and incubated at 37°C in 5% CO_2_ for 2 days without cycloheximide or penicillin. The infected cells from each well were harvested individually by scraping cells with a 1 ml filter tip and then lysed by vortexing with glass beads. The cell debris was removed by spinning at 1000 rpm for 5 min. The supernatant was saved (∼2 ml) and mixed with 2 ml of 4SP and stored in −80°C freezer (this was called T_0_).

5. Passage and selection. The T_0_ inocula were used to infect McCoy cells in a T_75_ flask (passage 1). Potential transformants were grown in medium containing cycloheximide (1 µg/ml) and selected with 10 units/ml of penicillin G (Sigma product no. P3032). Under these conditions, most inclusions were large and vacuolar as previously described [6]. *Chlamydia* were grown for two days before harvesting as ‘T_1_’ and this was used to infect McCoy cells as passage 2 in a T_25_ flask and selected with 10 units/ml of penicillin. Passaging was continued for 2–4 times in T_25_ flasks with 10 units/ml of penicillin until only normal inclusions were recovered. The passage and selection procedures are summarized in Table S1.

### Genomic DNA extraction from *C. trachomatis* strains

The genomic DNA of *C. trachomatis* L2, *C. trachomatis* L2 (25667R) and pBR325::L2- transformed *C. trachomatis* L2 (or L2, L2P- and L2/pBR325::L2 in short) were extracted from *Chlamydia* inocula (collected from infected McCoy cells in T_75_ flasks) using Wizard Genomic DNA Purification Kit (Promega, Cat. No. A1120). The genomic DNA of pGFP::SW2- transformed *C. trachomatis* L2 and transformed *C. trachomatis* L2 (25667R) (or L2/pGFP::SW2 and L2P-/pGFP::SW2 in short) was extracted from *Chlamydia* inocula (collected from a well of infected McCoy cells in 6-well tray) using NucleoSpin Tissue (Fisher Scientific, Cat. No. NZ74095250). The genomic DNA extracted from transformed *C. trachomatis* (L2/pBR325::L2, L2/pGFP::SW2 or L2P-/pGFP::SW2) were used for sequencing and the transformation of *E. coli* to recover the shuttle plasmids.

### Southern blotting

Restriction endonuclease digests of chlamydial genomic DNA were separated on agarose gels and then transferred to membranes using standard techniques [26]. The DNA used as probes in Figure 2 were either a ∼550 bp PCR amplicon for the β-lactamase gene using primer pair AmpF 5′-TTACCAATGCTTAAT-3′ and AmpR 5′-TACTCACCAGACACAG-3′) using pBR325::L2 as template or the whole recombinant chlamydial plasmid (pL2) released from the cloning vector pBR325::L2 by complete digestion with *Bam* HI (the insert was separated from the cloning vector by gel electrophoresis and the 7.5 kb fragment then eluted). DNA fragments were labeled with [α-^32^P]deoxy-CTP using a random primer labeling kit (Promega). The labeled products were purified by gel filtration on Sephadex G50. Membranes were pre-hybridized, hybridized overnight and then washed according to the manufacturer's standard conditions at 65°C. Dried membranes were exposed to Kodak XAR-5 film. In Figure 7 blots were probed with nonradioactive, digoxigenin-11-dUTP-labeled probes (Random primed DNA labelling) and chemiluminescence detection with CSPD (Roche Diagnostics Ltd., Product No. 11 585 614 910). The *gfp* probe was a 739 bp *Bam* HI/*Bgl* II fragment from pGFP::SW2, which annealed to a 1445 bp fragment of *Bam* HI digested pGFP::SW2 or a 3625 bp fragment of *Bgl* II digested pGFP::SW2. The pSW2 probe template was a 2518 bp *Eco* RI fragment from pGFP::SW2 (between pSW2 CDS4 and CDS7), which annealed to a 7169 bp fragment of *Bam* HI digested pGFP::SW2 or a 5555 bp fragment of *Bgl* II digested pGFP::SW2. The *ompA* probe template was a 1022 bp PCR product from *C. trachomatis* L2 (25667R) using primers PCTM3 (5′- TCCTTGCAAGCTCTGCCTGTGGGGAATCCT-3′) and NR1 (5′-CCGCAAGATTTTCTAGATTTC-3′) based on the *C. trachomatis* L2/434/Bu *ompA* sequence, this probe annealed to a 8837 bp fragment of *Bam* HI digested genomic DNA or a 5518 bp fragment of *Bgl* II digested genomic DNA.

### pBR325::L2 copy number determination

McCoy cells grown to confluence in 96 well trays were infected with *C. trachomatis* L2/434/Bu transformed by pBR325::L2 at MOI  = 1.0. EBs were allowed to adsorb to cells for 1 h at 37°C; cells were then washed with PBS to remove any residual unadsorbed EBs. The infected cells (performed in quadruplicate) were overlaid with 100 µl culture medium and incubated at 37°C in 5% CO_2_. At 65 h post infection, when the developmental cycle had completed, samples were stored (after snap freezing) at −80°C. For penicillin - treated cultures, medium containing 10 units/ml penicillin G was added at the time of infection. Chromosomal and plasmid DNA was extracted in a microplate format following a well described protocol. The residue was then resuspended in 100 µl nuclease-free water. Samples were diluted 1 in 100 prior to quantitative real time polymerase chain reaction (qPCR) analysis. A quantitative real-time PCR protocol was used to determine the absolute number of chlamydial plasmids and genomes in samples using 5′- exonuclease (TaqMan) assays with unlabelled primers andcarboxyfluorescein/carboxytetramethylrhodamine (FAM/TAMRA) dual-labeled probes as has been described previously.

### Immunoblotting for detection of β-lactamase, chloramphenicol acetyl transferase and green fluorescent protein

Proteins from purified EBs and RBs were run on 10% SDS PAGE gels and the proteins were electroblotted onto a BioRad Immun-Blot PVDF (polyvinylidene difluoride) membrane for 1 h at 15 V. The membrane was incubated in a solution of PBS supplemented with 0.05% Tween-20 (PBS-T) and 5% dried milk for 30 min at room temperature. Commercially available antibodies that recognize the enzymes β-lactamase (AbCam mouse monoclonal Cat. No. ab12251) and chloramphenicol acetyl transferase (Sigma anti-CAT antiserum from rabbit product no. C9336) were used at a concentration of 10 µg/ml, the anti-GFP mouse monoclonal antibodies (Roche Cat no. 11814 460 001) were used at 0.4 µg/ml in a solution of PBS-T supplemented with 1% dried milk, and incubated with the membrane for 1 h at RT. Following extensive washing with PBS-T the membrane was incubated for 1 h at room temperature with horseradish peroxidase-conjugated goat- anti-mouse or goat- anti-rabbit antibody (BioRad cat nos. 172-1011 and 172-1019) at the recommended dilution. After further washing with PBS-T the membrane was incubated in Pierce ECL Western Blotting Substrate (Thermo Scientific product no. 32106) as described by the manufacturer's instructions and exposed to Kodak BioMax XAR film.

### Nitrocefin assays for β-lactamase

Nitrocefin changes from yellow to red in the presence of β-lactamase, therefore it can be used as an indicator of β-lactamase activity. A standard Nitrocefin assay was set up in 1 M Phosphate buffer (K_2_HPO_4_.3H_2_0; KH_2_PO_4_) pH 7.0 using Nitrocefin (0.5 mg/ml) as described by the manufacturer (Calbiochem, Darmstadt, Germany). Equal amounts of gradient-purified *C. trachomatis* RBs (as determined by qPCR assay) from pBR325::pL2- transformed *C. trachomatis* L2/434/Bu and the wild–type parental *C. trachomatis* L2/434/Bu, and *E. coli* and pBR325::pL2- transformed *E. coli* were used in this assay and samples were taken at 5 min intervals for OD readings at 486 nm.

### Time course of infection

McCoy cells grown to confluence in 24 well trays were infected with *C. trachomatis* at MOI = 1. EBs were allowed to adsorb to cells for 1 h at 37°C and the infected cells were washed with PBS to remove any non-adsorbed EBs. The cells were then overlaid with culture medium or culture medium containing penicillin G at 10 units/ml and incubated at 37°C in 5% CO_2._ The infection was stopped at 8-hourly time points (8, 16, 24, 32, 40, 48, 56, 64 and 72 h) by scraping up the cells and vortexing with glass beads. The samples were then rapidly frozen and stored at −80°C.

### Iodine staining of inclusions

*C. trachomatis* strains were cultured in McCoy cells on coverslips. Briefly, infected cells bearing *C. trachomatis* inclusions were washed with PBS and then fixed to coverslips with ice-cold methanol. The coverslips were stained with 5% iodine stain (containing both potassium iodide and iodine in 50% ethanol) for 10 min. The stain was then changed for 2.5% iodine stain for 10 min and mounted in 5% iodine stain in glycerol (1:1) for photomicroscopy.

## Supporting Information

Figure S1 **Quantification of plasmid and chromosomal copies in pBR325::L2- transformed** ***C. trachomatis*** **L2/434/Bu.** The *Chlamydia* were grown in the absence (-PEN) and presence of penicillin (+PEN at 10 units/ml). The graph shows the absolute number of chromosomal and plasmid DNA copies in cultures from 96 well plates harvested at 64 h post-infection (y – axis). These data were generated by qPCR from four separate experiments and the standard error bars are indicated. The plasmid copy number (16 – 19) is similar under selective and non-selective conditions.
(TIF)

Figure S2 **Effects of penicillin at 48 h post infection by transmission electron microscopy.** Mature *C. trachomatis* L2/434/Bu inclusions at 48 h post infection are shown in the absence (A) and presence (B) of penicillin (10 units/ml). Mature *C. trachomatis* L2/434/Bu transformed by pBR325::L2 inclusions at 48 h post infection are shown in the absence (C) and presence (D) of penicillin. *C. trachomatis* L2/434/Bu transformed by pBR325::L2 is unaffected by penicillin treatment. The scale bar represents 5 µm, all electron micrographs were taken at the same magnification.
(TIF)

Figure S3 **Plasmid pBR325::L2 features and sequence.** The original pBR325::L2plasmid sequence (13495 bp) was based on GenBank data. It was created by ligating the L2 plasmid *Bam* HI fragment (7499 bp, GenBank: X07547) into the pBR325 cloning vector (5996 bp, GenBank: L08855) *Bam* HI site. The whole plasmid was sequenced from total DNA extracted from pBR325::L2 transformed *C. trachomatis* L2 and also from plasmid DNA recovered from *E. coli*. The changes from the GenBank sequence were two nucleotide substitutions, two deletions and one insertion, these changes are all located to the pBR325 vector in non-coding regions, hence the actual plasmid is one nucleotide shorter than the predicted sequence.
(DOC)

Figure S4 **Expression of β-lactamase and chloramphenicol acetyl transferase by EBs and RBs in transformed** ***C. trachomatis*** **.** Equal amounts of proteins from purified EBs and RBs from *C. trachomatis* L2/434/Bu (tracks 1 and 2) and *C. trachomatis* L2/434/Bu transformed by pBR325::L2 (tracks 3 and 4) were separated by SDS PAGE and immunoblotted with antisera to β-lactamase panel A and chloramphenicol acetyl transferase panel B. The two arrows in panel A indicate the mature and unprocessed forms of β-lactamase. The single arrow in panel B indicates the presence of chloramphenicol acetyl transferase.
(TIF)

Figure S5 **Plasmid pRSGFPCAT features and sequence.** The plasmid pRSGFPCAT is a small in house vector (2670 bp) containing the pUC origin and the RSGFP-CAT gene-fusion under the control of the meningococcal class I protein promoter (MCIP, designated nmP in Figure 5) from MC50. It was constructed by multiple cloning steps. Individual fragments were generated by PCR (see the table below for the sources of each fragment).
(DOC)

Figure S6 **Map of the vectors used as a basis for constructing pGFP::SW2.** Chlamydial plasmid pSW2 (A - dark blue) was ligated into the *Bam* HI site of pSP73 (B - red) to give intermediate plasmid pSP73::SW2 (C). The *Pst* I – *Sal* I fragment (light blue) from in-house vector pRSGFPCAT (D) was ligated in to *Pst* I/*Sal* I cleaved pSP73::SW2 to give the final vector pGFP::SW2 (E).
(TIF)

Figure S7 **Plasmid pGFP::SW2 features and sequence.** The pGFP::SW2 (11539 bp) was created by inserting the *C. trachomatis* SW2 plasmid (7169 bp, GenBank: FM865439.1) *Bam* HI fragment into the pSP73 cloning vector (2464 bp, GenBank: X65333.2) *Bam* HI site (forming an intermediate construct pSP73::SW2), then inserting the *Pst* I/*Sal* I fragment (RSGFPCAT cassette) from pRSGFPCAT (2670 bp, to be deposited in GenBank) into *Pst* I/*Sal* I sites of pSP73::SW2 (on pSP73 backbone). The promoter for GFPCAT expression is the meningococcal class I protein promoter (MCIP, designated nmP in Figure 5) for outer membrane protein PorA from *Neisseria meningitidis* MC50. The plasmids pRSGFPCAT and pSW2, and the sequences around cloning sites of pGFP::SW2 were verified.
(DOC)

Figure S8 **Expression of GFPCAT in** ***E. coli*** **and** ***C. trachomatis*** **L2/434/Bu transformed by pGFP::SW2.** Protein samples of *Chlamydia* lysates and *E. coli* lysates were separated by 10% SDS PAGE gel and immunoblotted with anti-GFP monoclonal antibodies (Roche, Cat. # 11 814 460 001). Tracks 1 and 2 are *C. trachomatis* L2/434/Bu and *C. trachomatis* L2/434/Bu transformed by pGFP::SW2. Tracks 3 and 4 are *E.coli* and the same *E.coli* strain transformed by pGFP::SW2. The GFPCAT fusion protein is arrowed at 53kDa.
(TIF)

Table S1 Summary of conditions to select penicillin-resistant *C. trachomatis* transformants
(DOC)
