# Supplementary figures and images for: Development of a Transformation System for Chlamydia trachomatis: Restoration of Glycogen Biosynthesis by Acquisition of a Plasmid Shuttle Vector
Source: PLoS Pathog. 2011 Sep 22;7(9):e1002258. doi: 10.1371/journal.ppat.1002258 (PMC3178582; doi:10.1371/journal.ppat.1002258)

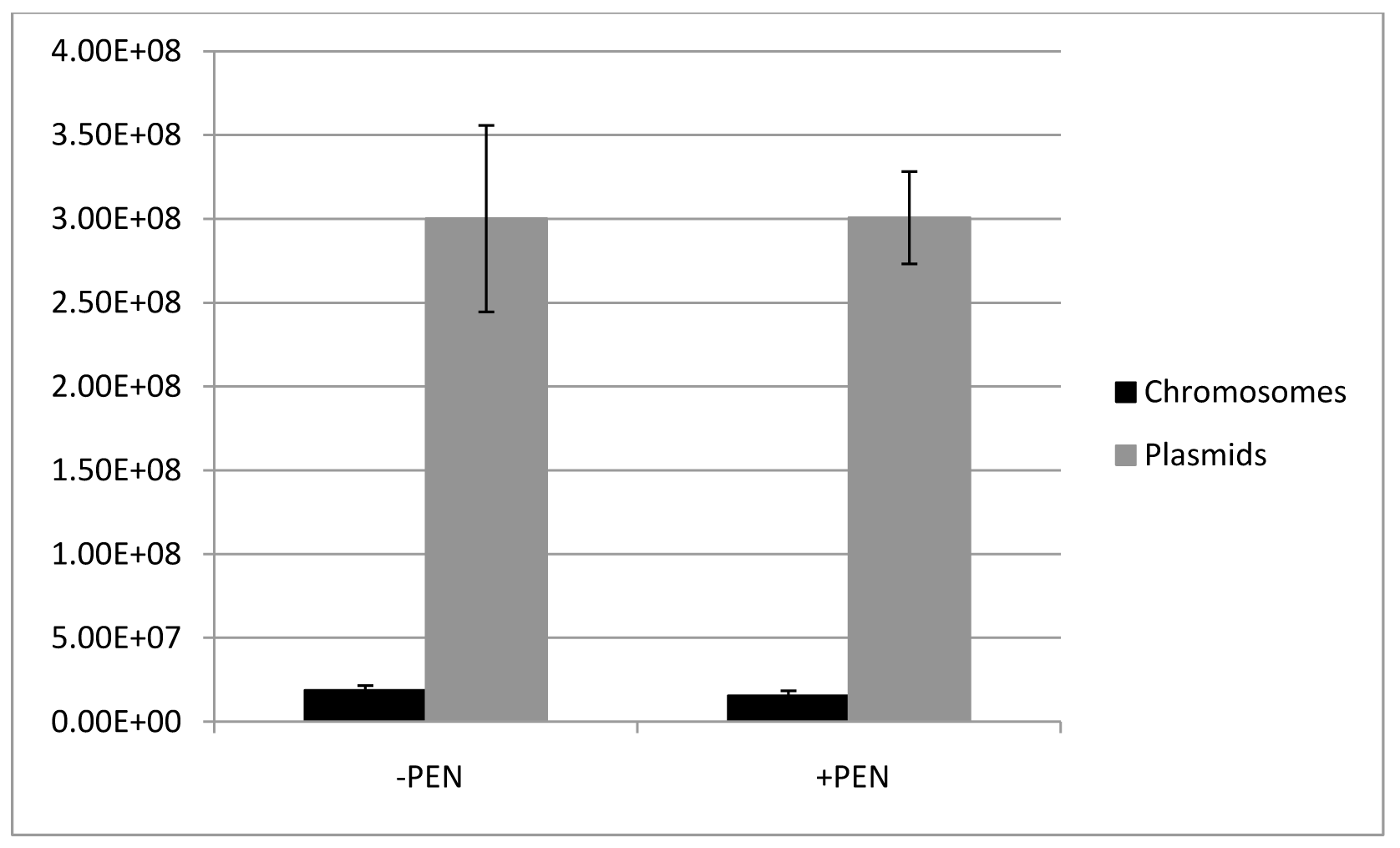

Supplement: Figure S1 — Quantification of plasmid and chromosomal copies in pBR325::L2- transformed C. trachomatis L2/434/Bu. The Chlamydia were grown in the absence (-PEN) and presence of penicillin (+PEN at 10 units/ml). The graph shows the absolute number of chromosomal and plasmid DNA copies in cultures from 96 well plates harvested at 64 h post-infection (y – axis). These data were generated by qPCR from four separate experiments and the standard error bars are indicated. The plasmid copy number (16 – 19) is similar under selective and non-selective conditions. (TIF) [file ppat.1002258.s001.tif]

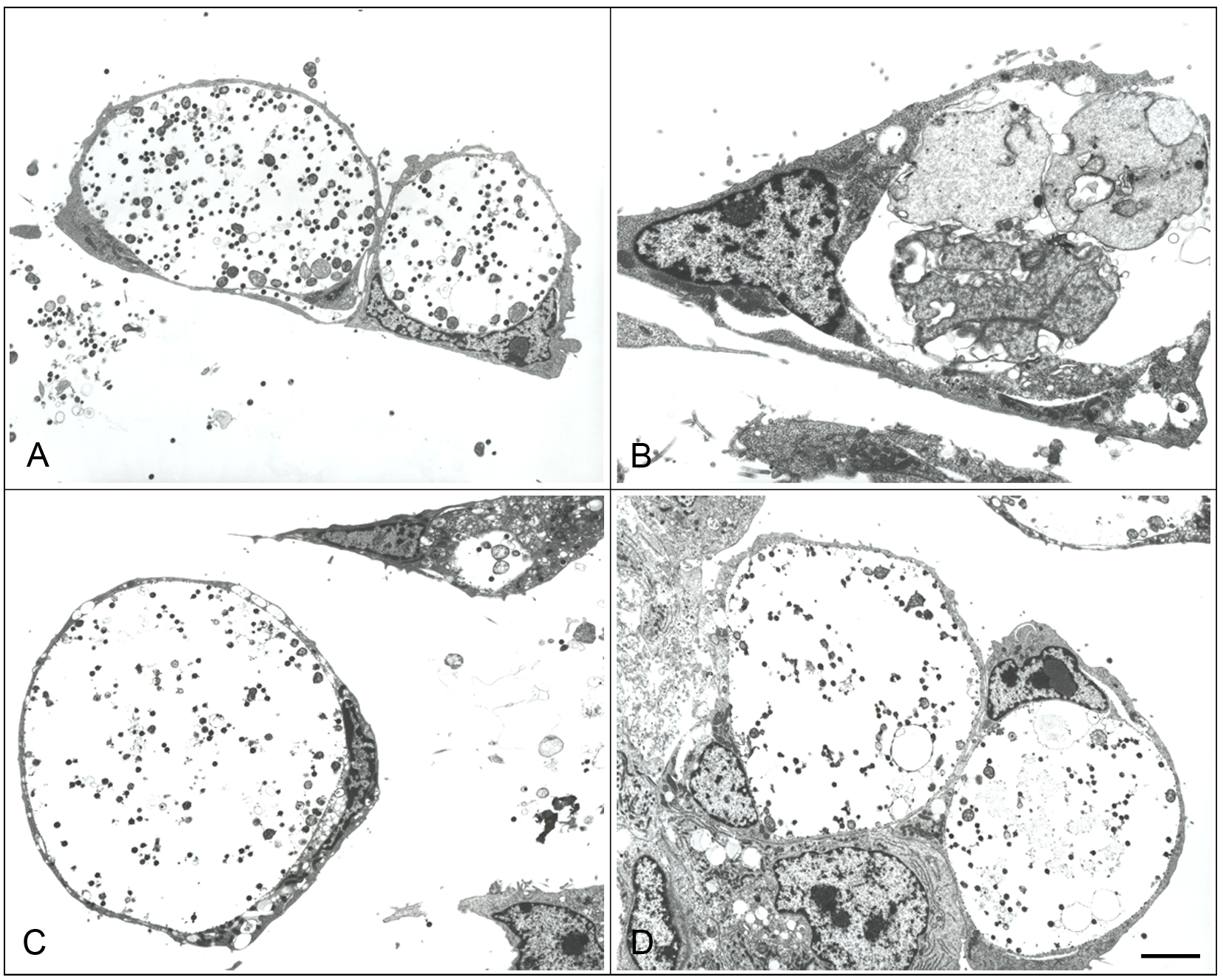

Supplement: Figure S2 — Effects of penicillin at 48 h post infection by transmission electron microscopy. Mature C. trachomatis L2/434/Bu inclusions at 48 h post infection are shown in the absence (A) and presence (B) of penicillin (10 units/ml). Mature C. trachomatis L2/434/Bu transformed by pBR325::L2 inclusions at 48 h post infection are shown in the absence (C) and presence (D) of penicillin. C. trachomatis L2/434/Bu transformed by pBR325::L2 is unaffected by penicillin treatment. The scale bar represents 5 µm, all electron micrographs were taken at the same magnification. (TIF) [file ppat.1002258.s002.tif]

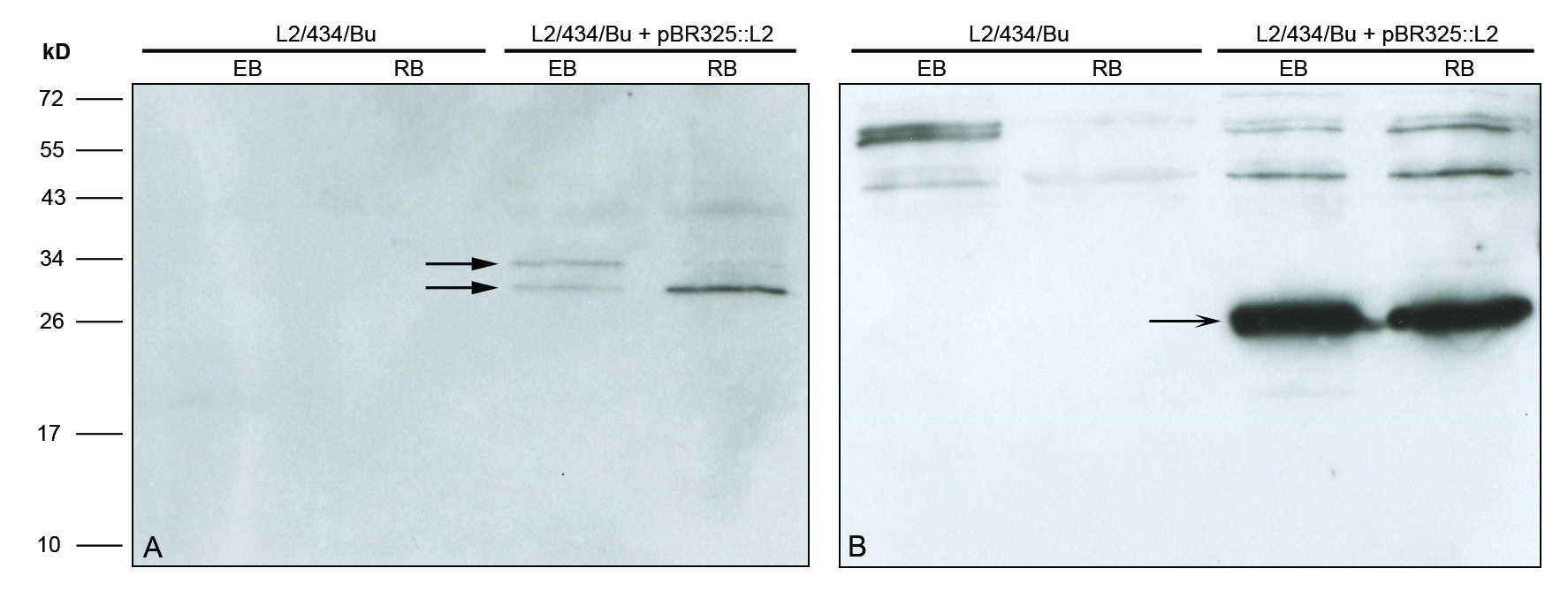

Supplement: Figure S4 — Expression of β-lactamase and chloramphenicol acetyl transferase by EBs and RBs in transformed C. trachomatis . Equal amounts of proteins from purified EBs and RBs from C. trachomatis L2/434/Bu (tracks 1 and 2) and C. trachomatis L2/434/Bu transformed by pBR325::L2 (tracks 3 and 4) were separated by SDS PAGE and immunoblotted with antisera to β-lactamase panel A and chloramphenicol acetyl transferase panel B. The two arrows in panel A indicate the mature and unprocessed forms of β-lactamase. The single arrow in panel B indicates the presence of chloramphenicol acetyl transferase. (TIF) [file ppat.1002258.s004.tif]

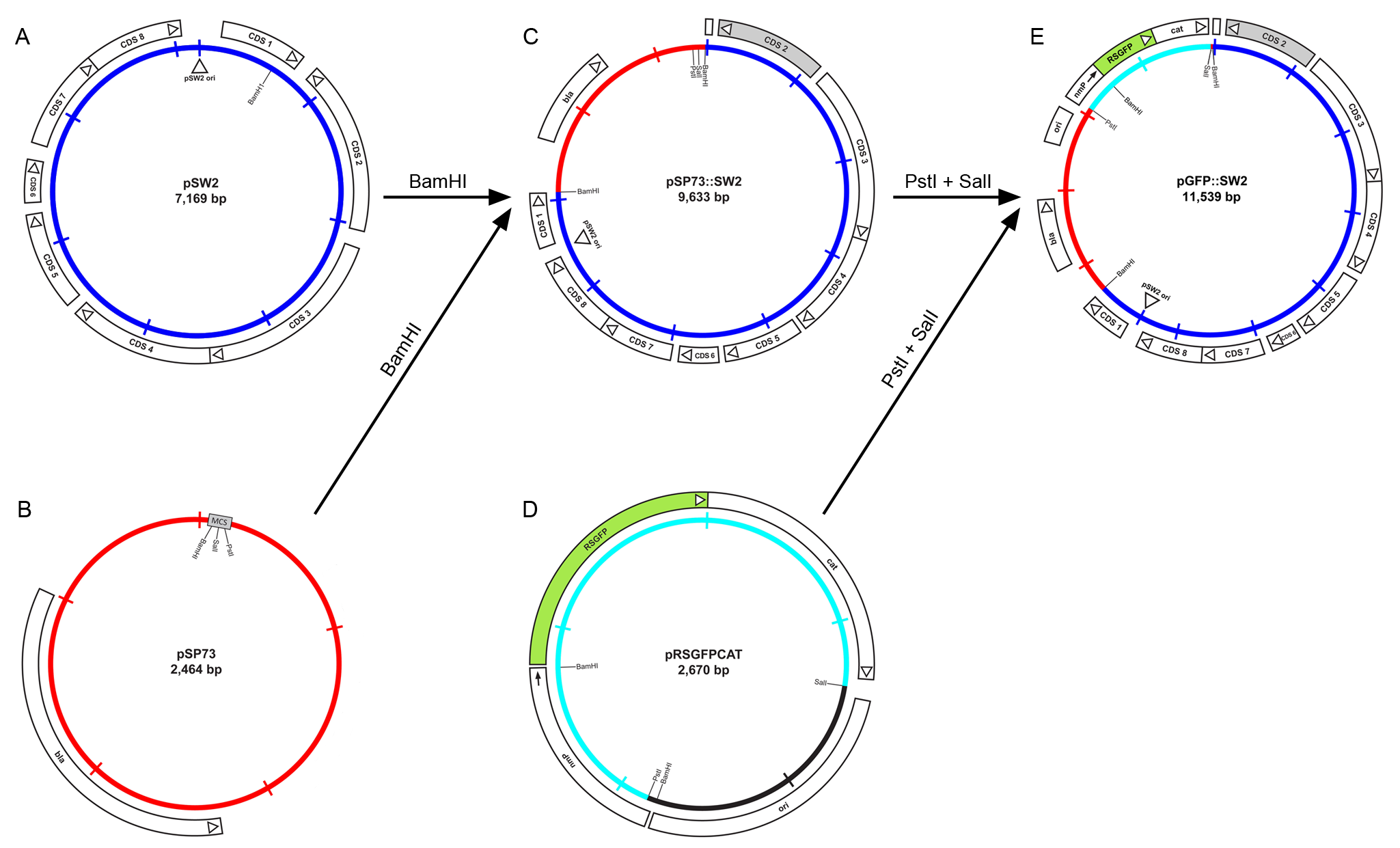

Supplement: Figure S6 — Map of the vectors used as a basis for constructing pGFP::SW2. Chlamydial plasmid pSW2 (A - dark blue) was ligated into the Bam HI site of pSP73 (B - red) to give intermediate plasmid pSP73::SW2 (C). The Pst I – Sal I fragment (light blue) from in-house vector pRSGFPCAT (D) was ligated in to Pst I/Sal I cleaved pSP73::SW2 to give the final vector pGFP::SW2 (E). (TIF) [file ppat.1002258.s006.tif]

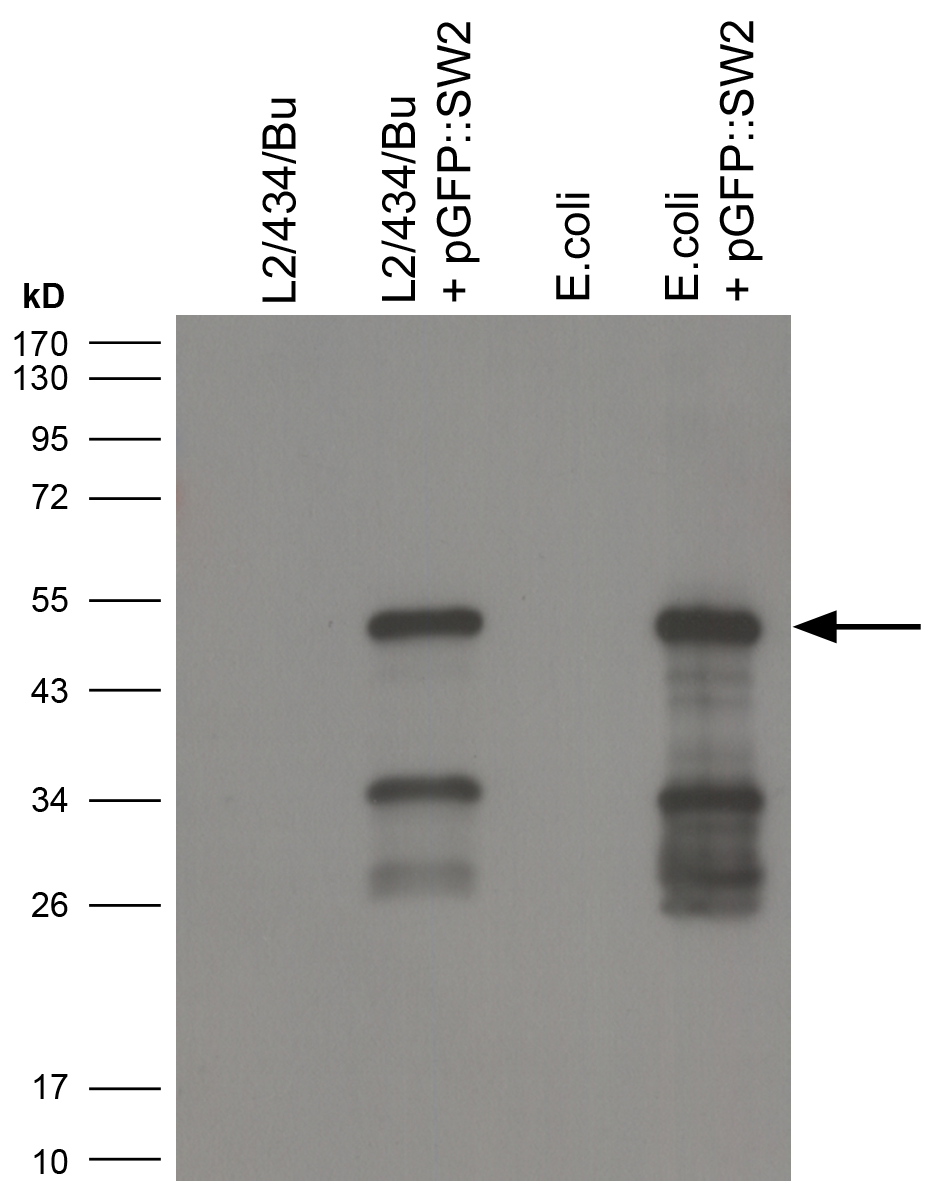

Supplement: Figure S8 — Expression of GFPCAT in E. coli and C. trachomatis L2/434/Bu transformed by pGFP::SW2. Protein samples of Chlamydia lysates and E. coli lysates were separated by 10% SDS PAGE gel and immunoblotted with anti-GFP monoclonal antibodies (Roche, Cat. # 11 814 460 001). Tracks 1 and 2 are C. trachomatis L2/434/Bu and C. trachomatis L2/434/Bu transformed by pGFP::SW2. Tracks 3 and 4 are E.coli and the same E.coli strain transformed by pGFP::SW2. The GFPCAT fusion protein is arrowed at 53kDa. (TIF) [file ppat.1002258.s008.tif]
