# Supplementary material for: Development of a Transformation System for Chlamydia trachomatis: Restoration of Glycogen Biosynthesis by Acquisition of a Plasmid Shuttle Vector
Source: PLoS Pathog. 2011 Sep 22;7(9):e1002258. doi: 10.1371/journal.ppat.1002258 (PMC3178582; doi:10.1371/journal.ppat.1002258)
Supplement: Figure S3 — Plasmid pBR325::L2 features and sequence. The original pBR325::L2plasmid sequence (13495 bp) was based on GenBank data. It was created by ligating the L2 plasmid Bam HI fragment (7499 bp, GenBank: X07547) into the pBR325 cloning vector (5996 bp, GenBank: L08855) Bam HI site. The whole plasmid was sequenced from total DNA extracted from pBR325::L2 transformed C. trachomatis L2 and also from plasmid DNA recovered from E. coli. The changes from the GenBank sequence were two nucleotide substitutions, two deletions and one insertion, these changes are all located to the pBR325 vector in non-coding regions, hence the actual plasmid is one nucleotide shorter than the predicted sequence. (DOC) [file ppat.1002258.s003.doc]

**Plasmid pBR325::L2 features and sequence**

| **Position on pBR325::L2**  **(sequence verified)** | **Feature** | **Length** | **Source** | **GenBank #** |
| --- | --- | --- | --- | --- |
| 1-7499 | pL2 *Bam* HI fragment | 7499 bp | pL2 | X07547 |
| 7500-13494 | pBR325 *Bam* HI fragment | 5995 bp | pBR325 | L08855 |
|  |  |  |  |  |
| 1478🡪486 | CDS2 | 993 bp | pL2 |  |
| 9310🡪8651 | *cat* | 660 bp | pBR325 |  |
| 9721🡪10581 | *bla* | 861 bp | pBR325 |  |
| 10726🡪11399 | pUC ori | 674 bp | pBR325 |  |

**The sequence of pBR325::L2 (13494bp) (sequence verified)**

1 GATCCGTAAG TTAGACGAAA TTTTGTCTTT GCGCACAGAC GATCTATTTT TTGCATCCAA TCAGATTTCC TTTCGCATTA AAAAAAGACA GAATAAAGAA

101 ACCAAAATTC TAATCACATT TCCTATCAGC TTAATGGAGG AGTTGCAAAA ATACACTTGT GGGAGAAATG GGAGAGTATT TGTTTCTAAA ATAGGGATTC

201 CTGTAACAAC AAGTCAGGTT GCGCATAATT TTAGGCTTGC AGAGTTCTAT AGTGCTATGA AAAAAAAATT ACTCCTAGAG TACTTCGTGC AAGCGCTTTG

301 ATTCATTTAA AGCAAATAGG ATTAAAAGAT GAGGAAATCA TGCGTATTTC CTGTCTTTCA TCGAGACAAA GTGTGTGTTC TTATTGTTCT GGGGAAGAGG

401 TAAGTCCTCT AGTACAAACA CCCCCAATAT TGTGATATAA TTAAAATTAT ATTCATATTC TGTTGCCAGA AAAAACACTT TTAGGCTATA TTAGAGCCAT

501 CTTCTTTGAA GCGTTGTCTT CTCGAGAAGA TTTATCGTAC GCAAATATCA TCTTTGCGGT TGCGTGTCCT GTGACCTTCA TTATGTCGGA GTCTGAGCAC

601 CCTAGGCGTT TGTACTCCGT CACAGCGGTT GCTCGAAGCA CGTGCGGGGT TATCTTAAAA GGGATTGCAG CTTGTAGTCC TGCTTGAGAG AACGTGCGGG

701 CGATTTGCCT TAACCCCACC ATTTTTCCGG AGCGAGTTAC GAAGACAAAA CCTCTTCGTT GACCGATGTA CTCTTGTAGA AAGTGCATAA ACTTCTGAGG

801 ATAAGTTATA ATAATCCTCT TTTCTGTCTG ACGGTTCTTA AGCTGGGAGA AAGAAATGGT AGCTTGTTGG AAACAAATCT GACTAATCTC CAAGCTTAAG

901 ACTTCAGAGG AGCGTTTACC TCCTTGGAGC ATTGTCTGGG CGATCAACCA ATCCCGGGCA TTGATTTTTT TTAGCTCTTT TAGGAAGGAC GCTGTTTGCA

1001 AACTGTTCAT CGCATCTGTT TTTACTATTT CCCTGGTTTT AAAAAATGTT CGACTATTTT CTTGTTTAGA AGGTTGCGCT ATAGCGACTA TTCCTTGAGT

1101 CATCCTGTTT AGGAATCTTG TTAAGGAAAT ATAGCTTGCT GCTCGAACTT GTTTAGTACC TTCGGTCCAA GAAGTCTTGG CAGAGGAAAC TTTTTTAATC

1201 GCATCTAGAA TTAGATTATG ATTTAAAAGG GAAAACTCTT GCAGATTCAT ATCCAAGGAC AATAGACCAA TCTTTTCTAA AGACAAAAAA GATCCTCGAT

1301 ATGATCTACA AGTATGTTTG TTGAGTGATG CGGTCCAATG CATAATAACT TCGAATAAGG AGAAGCTTTT CATGCGTTTC CAATAGGATT CTTGGCGAAT

1401 TTTTAAAACT TCCTGATAAG ACTTTTCGCT ATATTCTAAC GACATTTCTT GCTGCAAAGA TAAAATCCCT TTACCCATGA AATCCCTCGT GATATAACCT

1501 ATCCGTAAAA TGTCCTGATT AGTGAAATAA TCAGGTTGTT AACAGGATAG CACGCTCGGT ATTTTTTTAT ATAAACATGA AAACTCGTTC CGAAATAGAA

1601 AATCGCATGC AAGATATCGA GTATGCGTTG TTAGGTAAAG CTCTGATATT TGAAGACTCT ACTGAGTATA TTCTGAGGCA GCTTGCTAAT TATGAGTTTA

1701 AGTGTTCTCA TCATAAAAAC ATATTCATAG TATTTAAATA CTTAAAAGAC AATGGATTAC CTATAACTGT AGACTCGGCT TGGGAAGAGC TTTTGCGGCG

1801 TCGTATCAAA GATATGGACA AATCGTATCT CGGGTTAATG TTGCATGATG CTTTATCAAA TGACAAGCTT AGATCCGTTT CTCATACGGT TTTCCTCGAT

1901 GATTTGAGCG TGTGTAGCGC TGAAGAAAAT TTGAGTAATT TCATTTTCCG CTCGTTTAAT GAGTACAATG AAAATCCATT GCGTAGATCT CCGTTTCTAT

2001 TGCTTGAGCG TATAAAGGGA AGGCTTGACA GTGCTATAGC AAAGACTTTT TCTATTCGCA GCGCTAGAGG CCGGTCTATT TATGATATAT TCTCACAGTC

2101 AGAAATTGGA GTGCTGGCTC GTATAAAAAA AAGACGAGCA ACGTTCTCTG AGAATCAAAA TTCTTTCTTT GATGCCTTCC CAACAGGATA CAAGGATATT

2201 GATGATAAAG GAGTTATCTT AGCTAAAGGT AATTTCGTGA TTATAGCAGC TAGGCCATCT ATAGGGAAAA CTGCTTTAGC TATAGACATG GCGATAAATC

2301 TTGCGGTTAC TCAACAGCGT AGAGTTGGTT TCCTATCTCT AGAAATGAGC GCAGGTCAAA TTGTTGAGCG GATTATTGCT AATTTAACAG GAATATCTGG

2401 TGAAAAATTA CAAAGAGGGG ATCTCTCTAA AGAAGAATTA TTCCGAGTAG AAGAAGCTGG AGAAACAGTT AGAGAATCAC ATTTTTATAT CTGCAGTGAT

2501 AGTCAGTATA AGCTTAATTT AATCGCGAAT CAGATCCGGT TGCTGAGAAA AGAAGATCGA GTAGACGTAA TATTTATCGA TTACTTGCAG TTGATCAACT

2601 CATCGGTTGG AGAAAATCGT CAAAATGAAA TAGCAGATAT ATCTAGAACC TTAAGAGGTT TAGCCTCAGA GCTAAACATT CCTATAGTTT GTTTATCCCA

2701 ACTATCTAGA AAAGTTGAGG ATAGAGCAAA TAAAGTTCCC ATGCTTTCAG ATTTGCGAGA CAGCGGTCAA ATAGAGCAAG ACGCAGATGT GATTTTGTTT

2801 ATCAATAGGA AGGAATCGTC TTCTAATTGT GAGATAACTG TTGGGAAAAA TAGACATGGA TCGGTTTTCT CTTCGGTATT ACATTTCGAT CCAAAAATTA

2901 GTAAATTCTC CGCTATTAAA AAAGTATGGT AAATTATAGT AACTGCCACT TCATCAAAAG TCCTATCCAC CTTGAAAATC AGAAGTTTGG AAGAAGACCT

3001 GGTCAATCTA TTAAGATATC TCCCAAATTG GCTCAAAATG GGATGGTAGA AGTTATAGGT CTTGATTTTC TTTCATCTCA TTACCATGCA TTAGCAGCTA

3101 TCCAAAGATT GCTGACTGCA ACGAATTACA AGGGGAACAC AAAAGGGGTT GTTTTATCCA GAGAATCAAA TAGTTTTCAA TTTGAAGGAT GGATACCAAG

3201 AATCCGTTTT ACAAAAACTG AATTCTTAGA GGCTTATGGA GTTAAGCGGT ATAAAACATC CAGAAATAAG TATGAGTTTA GTGGAAAAGA AGCTGAAACT

3301 GCTTTAGAAG CCTTATACCA TTTAGGACAT CAACCGTTTT TAATAGTGGC AACTAGAACT CGATGGACTA ATGGAACACA AATAGTAGAC CGTTACCAAA

3401 CTCTTTCTCC GATCATTAGG ATTTACGAAG GATGGGAAGG TTTAACTGAC GAAGAAAATA TAGATATAGA CTTAACACCT TTTAATTCAC CATCTACACG

3501 GAAACATAAA GGGTTCGTTG TAGAGCCATG TCCTATCTTG GTAGATCAAA TAGAATCCTA CTTTGTAATC AAGCCTGCAA ATGTATACCA AGAAATAAAA

3601 ATGCGCTTCC CAAATGCATC AAAGTATGCT TACACATTTA TCGACTGGGT GATTACAGCA GCTGCGAAAA AGAGACGAAA ATTAACTAAG GATAATTCTT

3701 GGCCAGAAAA CTTGTTCTTA AACGTTAACG TTAAAAGTCT TGCATATATT TTAAGGATGA ATCGGTACAT TTGTACAAGG AACTGGAAAA AAATCGAGTT

3801 AGCTATCGAT AAATGTATAG AAATCGCCAT TCAGCTTGGT TGGTTATCTA GAAGAAAACG CATTGAATTT CTGGATTCTT CTAAACTCTC TAAAAAAGAA

3901 ATTCTATATC TAAATAAAGA GCGTTTTGAA GAAATAACTA AGAAATCTAA AGAACAAATG GAACAATTAG AACAAGAATC TATTAATTAA TAGCAAACTT

4001 GAAACTAAAA ACCTAATTTA TTTAAAGCTC AAAATAAAAA AGAGTTTTAA AATGGGAAAT TCTGGTTTTT ATTTGTATAA CACTCAAAAC TGCGTCTTTG

4101 CTGATAATAT CAAAGTTGGG CAAATGACAG AGCCGCTCAA GGACCAGCAA ATAATCCTTG GGACAACATC AACACCTGTC GCAGCCAAAA TGACAGCTTC

4201 TGATGGAATA TCTTTAACAG TCTCCAATAA TCCATCAACC AATGCTTCTA TTACAATTGG TTTGGATGCG GAAAAAGCTT ACCAGCTTAT TCTAGAAAAG

4301 TTGGGAGATC AAATTCTTGG TGGAATTGCT GATACTATTG TTGATAGTAC AGTCCAAGAT ATTTTAGACA AAATCACAAC AGACCCTTCT CTAGGTTTGT

4401 TGAAAGCTTT TAACAACTTT CCAATCACTA ATAAAATTCA ATGCAACGGG TTATTCACTC CCAGGAACAT TGAAACTTTA TTAGGAGGAA CTGAAATAGG

4501 AAAATTCACA GTCACACCCA AAAGCTCTGG GAGCATGTTC TTAGTCTCAG CAGATATTAT TGCATCAAGA ATGGAAGGCG GCGTTGTTCT AGCTTTGGTA

4601 CGAGAAGGTG ATTCTAAGCC CTACGCGATT AGTTATGGAT ACTCATCAGG CGTTCCTAAT TTATGTAGTC TAAGAACCAG AATTATTAAT ACAGGATTGA

4701 CTCCGACAAC GTATTCATTA CGTGTAGGCG GTTTAGAAAG CGGTGTGGTA TGGGTTAATG CCCTTTCTAA TGGCAATGAT ATTTTAGGAA TAACAAATAC

4801 TTCTAATGTA TCTTTTTTGG AGGTAATACC TCAAACAAAC GCTTAAACAA TTTTTATTGG ATTTTTCTTA TAGGTTTTAT ATTTAGAGAA AAAAGTTCGA

4901 ATTACGGGGT TTGTTATGCA AAATAAAAGC AAAGTGAGGG ACGATTTTAT TAAAATTGTT AAAGATGTGA AAAAAGATTT CCCCGAATTA GACCTAAAAA

5001 TACGAGTAAA CAAGGAAAAA GTAACTTTCT TAAATTCTCC CTTAGAACTC TACCATAAAA GTGTCTCACT AATTCTAGGA CTGCTTCAAC AAATAGAAAA

5101 CTCTTTAGGA TTATTCCCAG ACTCTCCTGT TCTTGAAAAA TTAGAGGATA ACAGTTTAAA GCTAAAAAAG GCTTTGATTA TGCTTATCTT GTCTAGAAAA

5201 GACATGTTTT CCAAGGCTGA ATAGATAACT TACTCTAACG TTGGAGTTGA TTTGCACACC TTAGTTTTTT GCTCTTTTAA GGGAGGAACT GGAAAAACAA

5301 CACTTTCTCT AAACGTGGGA TGCAACTTGG CCCAATTTTT AGGGAAAAAA GTGTTACTTG CTGACCTAGA CCCGCAATCC AATTTATCTT CTGGATTGGG

5401 GGCTAGTGTC AGAAGTAACC AAAAAGGCTT ACACGACATA GTATACACAT CAAACGATTT AAAATCAATC ATTTGCGAAA CAAAAAAAGA TAGTGTGGAC

5501 CTAATTCCTG CATCATTTTT ATCCGAACAG TTTAGAGAAT TGGATATTCA TAGAGGACCT AGTAACAACT TAAAGTTATT TCTGAATGAG TACTGCGCTC

5601 CTTTTTATGA CATCTGCATA ATAGACACTC CACCTAGCCT AGGAGGGTTA ACGAAAGAAG CTTTTGTTGC AGGAGACAAA TTAATTGCTT GTTTAACTCC

5701 AGAACCTTTT TCTATTCTAG GGTTACAAAA GATACGTGAA TTCTTAAGTT CGGTCGGAAA ACCTGAAGAA GAACACATTC TTGGAATAGC TTTGTCTTTT

5801 TGGGATGATC GTAACTCGAC TAACCAAATG TATATAGACA TTATCGAGTC TATTTACAAA AACAAGCTTT TTTCAACAAA AATTCGTCGA GATATTTCTC

5901 TCAGCCGTTC TCTTCTTAAA GAAGATTCTG TAGCTAATGT CTATCCAAAT TCTAGGGCCG CAGAAGATAT TCTGAAGTTA ACGCATGAAA TAGCAAATAT

6001 TTTGCATATC GAATATGAAC GAGATTACTC TCAGAGGACA ACGTGAACAA ACTAAAAAAA GAAGCGAATG TCTTTTTTAA AAAAAATCAA ACTGCCGCTT

6101 CTTTAGATTT TAAGAAGACG CTTCCTTCCA TTGAACTATT CTCAGCAACT TTGAATTCTG AGGAAAGTCA GAGTTTGGAT CAATTATTTT TATCAGAGTC

6201 CCAAAACTAT TCGGATGAAG AATTTTATCA AGAAGACATC CTAGCGGTAA AACTGCTTAC TGGTCAGATA AAATCCATAC AGAAGCAACA CGTACTTCTT

6301 TTAGGAGAAA AAATCTATAA TGCTAGAAAA ATCCTGAGTA AGGATCACTT CTCCTCAACA ACTTTTTCAT CTTGGATAGA GTTAGTTTTT AGAACTAAGT

6401 CTTCTGCTTA CAATGCTCTT GCATATTACG AGCTTTTTAT AAACCTCCCC AACCAAACTC TACAAAAAGA GTTTCAATCG ATCCCCTATA AATCCGCATA

6501 TATTTTGGCC GCTAGAAAAG GCGATTTAAA AACCAAGGTC GATGTGATAG GGAAAGTATG TGGAATGTCG AACTCATCGG CGATAAGGGT GTTGGATCAA

6601 TTTCTTCCTT CATCTAGAAA CAAAGACGTT AGAGAAACGA TAGATAAGTC TGATTCAGAG AAGAATCGCC AATTATCTGA TTTCTTAATA GAGATACTTC

6701 GCATCATGTG TTCCGGAGTT TCTTTGTCCT CCTATAACGA AAATCTTCTA CAACAGCTTT TTGAACTTTT TAAGCAAAAG AGCTGATCCT CCGTCAGCTC

6801 ATATATATAT CTATTATATA TATATATTTA GGGATTTGAT TTTACGAGAG AGATTTGCAA CTCTTGGTGG TAGACTTTGC AACTCTTGGT GGTAGACTTT

6901 GCAACTCTTG GTGGTAGACT TTGCAACTCT TGGTGGTAGA CTTGGTCATA ATGGACTTTT GTTGAAAAAT TTCTTAAAAT CTTAGAGCTC CGATTTTGAA

7001 TAGCTTTGGT TAAGAAAATG GGCTCGATGG CTTTCCATAA AAGTAGGTTG TTCTTAACTT TTGGGGACGC GTCGGAAATT TGGTTATCTA CTTTATCTCA

7101 TCTAACTAGA AAAAATTATG CGTCTGGGAT TAACTTTCTT GTTTCTTTAG AGATTCTGGA TTTATCGGAA ACCTTGATAA AGGCTATTTC TCTTGACCAC

7201 AGCGAATCTT TGTTTAAAAT CAAGTCTCTA GATGTTTTTA ATGGAAAAGT CGTTTCAGAG GCCTCTAAAC AGGCTAGAGC GGCATGCTAC ATATCTTTCA

7301 CAAAGTTTTT GTATAGATTG ACCAAGGGAT ATATTAAACC CGCTATTCCA TTGAAAGATT TTGGAAACAC TACATTTTTT AAAATCCGAG ACAAAATCAA

7401 AACAGAATCG ATTTCTAAGC AGGAATGGAC AGTTTTTTTT GAAGCGCTCC GGATAGTGAA TTATAGAGAC TATTTAATCG GTAAATTGAT TGTACAAGGG

7501 ATCCACAGGA CGGGTGTGGT CGCCATGATC GCGTAGTCGA TAGTGGCTCC AAGTAGCGAA GCGAGCAGGA CTGGGCGGCG GCCAAAGCGG TCGGACAGTG

7601 CTCCGAGAAC GGGTGCGCAT AGAAATTGCA TCAACGCATA TAGCGCTAGC AGCACGCCAT AGTGACTGGC GATGCTGTCG GAATGGACGA TATCCCGCAA

7701 GAGGCCCGGC AGTACCGGCA TAACCAAGCC TATGCCTACA GCATCCAGGG TGACGGTGCC GAGGATGACG ATGAGCGCAT TGTTAGATTT CATACACGGT

7801 GCCTGACTGC GTTAGCAATT TAACTGTGAT AAACTACCGC ATTAAAGCTT ATCGATGATA AGCTGTCAAA CATGGCCTGT CGCTTGCGGT ATTCGGAATC

7901 TTGCACGCCC TCGCTCAAGC CTTCGTCACT GGTCCCGCCA CCAAACGTTT CGGCGAGAAG CAGGCCATTA TCGCCGGCAT GGCGGCCGAC GCGCTGGGCT

8001 ACGTCTTGCT GGCGTTCGCG ACGCGAGGCT GGATGGCCTT CCCCATTATG ATTCTTCTCG CTTCCGGCGG CATCGGGATG CCCGCGTTGC AGGCCATGCT

8101 GTCCAGGCAG GTAGATGACG ACCATCAGGG ACAGCTTCAA GGATCGCTCG CGGCTCTTAC CAGCCTAACT TCGATCATTG GACCGCTGAT CGTCACGGCG

8201 ATTTATGCCG CCTCGGCGAG CACATGGAAC GGGTTGGCAT GGATTGTAGG CGCCGCCCTA TACCTTGTCT GCCTCCCCGC GTTGCGTCGC GGTGCATGGA

8301 GCCGGGCCAC CTCGACCTGA ATGGAAGCCG GCGGCACCTC GCTAACGGAT TCACCGTTTT TATCAGGCTC TGGGAGGCAG AATAAATGAT CATATCGTCA

8401 ATTATTACCT CCACGGGGAG AGCCTGAGCA AACTGGCCTC AGGCATTTGA GAAGCACACG GTCACACTGC TTCCGGTAGT CAATAAACCG GTAAACCAGC

8501 AATAGACATA AGCGGCTATT TAACGACCCT GCCCTGAACC GACGACCGGG TCGAATTTGC TTTCGAATTT CTGCCATTCA TCCGCTTATT ATCACTTATT

8601 CAGGCGTAGC AACCAGGCGT TTAAGGGCAC CAATAACTGC CTTAAAAAAA TTACGCCCCG CCCTGCCACT CATCGCAGTA CTGTTGTAAT TCATTAAGCA

8701 TTCTGCCGAC ATGGAAGCCA TCACAAACGG CATGATGAAC CTGAATCGCC AGCGGCATCA GCACCTTGTC GCCTTGCGTA TAATATTTGC CCATGGTGAA

8801 AACGGGGGCG AAGAAGTTGT CCATATTGGC CACGTTTAAA TCAAAACTGG TGAAACTCAC CCAGGGATTG GCTGAGACGA AAAACATATT CTCAATAAAC

8901 CCTTTAGGGA AATAGGCCAG GTTTTCACCG TAACACGCCA CATCTTGCGA ATATATGTGT AGAAACTGCC GGAAATCGTC GTGGTATTCA CTCCAGAGCG

9001 ATGAAAACGT TTCAGTTTGC TCATGGAAAA CGGTGTAACA AGGGTGAACA CTATCCCATA TCACCAGCTC ACCGTCTTTC ATTGCCATAC GGAATTCCGG

9101 ATGAGCATTC ATCAGGCGGG CAAGAATGTG AATAAAGGCC GGATAAAACT TGTGCTTATT TTTCTTTACG GTCTTTAAAA AGGCCGTAAT ATCCAGCTGA

9201 ACGGTCTGGT TATAGGTACA TTGAGCAACT GACTGAAATG CCTCAAAATG TTCTTTACGA TGCCATTGGG ATATATCAAC GGTGGTATAT CCAGTGATTT

9301 TTTTCTCCAT TTTAGCTTCC TTAGCTCCTG AAAATCTCGA TAACTCAAAA AATACGCCCG GTAGTGATCT TATTTCATTA TGGTGAAAGT TGGAACCTCT

9401 TACGTGCCGA TCAACGTCTC ATTTTCGCCA AAAGTTGGCC CAGGGCTTCC CGGTATCAAC AGGGACACCA GGATTTATTT ATTCTGCGAA GTGATCTTCC

9501 GTCACAGGTA TTTATTCGAA GACGAAAGGG CCTCGTGATA CGCCTATTTT TATAGGTTAA TGTCATGATA ATAATGGTTT CTTAGACGTC AGGTGGCACT

9601 TTTCGGGGAA ATGTGCGCGG AACCCCTATT TGTTTATTTT TCTAAATACA TTCAAATATG TATCCGCTCA TGAGACAATA ACCCTGATAA ATGCTTCAAT

9701 AATATTGAAA AAGGAAGAGT ATGAGTATTC AACATTTCCG TGTCGCCCTT ATTCCCTTTT TTGCGGCATT TTGCCTTCCT GTTTTTGCTC ACCCAGAAAC

9801 GCTGGTGAAA GTAAAAGATG CTGAAGATCA GTTGGGTGCA CGAGTGGGTT ACATCGAACT GGATCTCAAC AGCGGTAAGA TCCTTGAGAG TTTTCGCCCC

9901 GAAGAACGTT TTCCAATGAT GAGCACTTTT AAAGTTCTGC TATGTGGCGC GGTATTATCC CGTGTTGACG CCGGGCAAGA GCAACTCGGT CGCCGCATAC

10001 ACTATTCTCA GAATGACTTG GTTGAGTACT CACCAGTCAC AGAAAAGCAT CTTACGGATG GCATGACAGT AAGAGAATTA TGCAGTGCTG CCATAACCAT

10101 GAGTGATAAC ACTGCGGCCA ACTTACTTCT GACAACGATC GGAGGACCGA AGGAGCTAAC CGCTTTTTTG CACAACATGG GGGATCATGT AACTCGCCTT

10201 GATCGTTGGG AACCGGAGCT GAATGAAGCC ATACCAAACG ACGAGCGTGA CACCACGATG CCTGCAGCAA TGGCAACAAC GTTGCGCAAA CTATTAACTG

10301 GCGAACTACT TACTCTAGCT TCCCGGCAAC AATTAATAGA CTGGATGGAG GCGGATAAAG TTGCAGGACC ACTTCTGCGC TCGGCCCTTC CGGCTGGCTG

10401 GTTTATTGCT GATAAATCTG GAGCCGGTGA GCGTGGGTCT CGCGGTATCA TTGCAGCACT GGGGCCAGAT GGTAAGCCCT CCCGTATCGT AGTTATCTAC

10501 ACGACGGGGA GTCAGGCAAC TATGGATGAA CGAAATAGAC AGATCGCTGA GATAGGTGCC TCACTGATTA AGCATTGGTA ACTGTCAGAC CAAGTTTACT

10601 CATATATACT TTAGATTGAT TTAAAACTTC ATTTTTAATT TAAAAGGATC TAGGTGAAGA TCCTTTTTGA TAATCTCATG ACCAAAATCC CTTAACGTGA

10701 GTTTTCGTTC CACTGAGCGT CAGACCCCGT AGAAAAGATC AAAGGATCTT CTTGAGATCC TTTTTTTCTG CGCGTAATCT GCTGCTTGCA AACAAAAAAA

10801 CCACCGCTAC CAGCGGTGGT TTGTTTGCCG GATCAAGAGC TACCAACTCT TTTTCCGAAG GTAACTGGCT TCAGCAGAGC GCAGATACCA AATACTGTCC

10901 TTCTAGTGTA GCCGTAGTTA GGCCACCACT TCAAGAACTC TGTAGCACCG CCTACATACC TCGCTCTGCT AATCCTGTTA CCAGTGGCTG CTGCCAGTGG

11001 CGATAAGTCG TGTCTTACCG GGTTGGACTC AAGACGATAG TTACCGGATA AGGCGCAGCG GTCGGGCTGA ACGGGGGGTT CGTGCACACA GCCCAGCTTG

11101 GAGCGAACGA CCTACACCGA ACTGAGATAC CTACAGCGTG AGCTATGAGA AAGCGCCACG CTTCCCGAAG GGAGAAAGGC GGACAGGTAT CCGGTAAGCG

11201 GCAGGGTCGG AACAGGAGAG CGCACGAGGG AGCTTCCAGG GGGAAACGCC TGGTATCTTT ATAGTCCTGT CGGGTTTCGC CACCTCTGAC TTGAGCGTCG

11301 ATTTTTGTGA TGCTCGTCAG GGGGGCGGAG CCTATGGAAA AACGCCAGCA ACGCGGCCTT TTTACGGTTC CTGGCCTTTT GCTGGCCTTT TGCTCACATG

11401 TTCTTTCCTG CGTTATCCCC TGATTCTGTG GATAACCGTA TTACCGCCTT TGAGTGAGCT GATACCGCTC GCCGCAGCCG AACGACCGAG CGCAGCGAGT

11501 CAGTGAGCGA GGAAGCGGAA GAGCGCCTGA TGCGGTATTT TCTCCTTACG CATCTGTGCG GTATTTCACA CCGCATATGG TGCACTCTCA GTACAATCTG

11601 CTCTGATGCC GCATAGTTAA GCCAGTATAC ACTCCGCTAT CGCTACGTGA CTGGGTCATG GCTGCGCCCC GACACCCGCC AACACCCGCT GACGCGCCCT

11701 GACGGGCTTG TCTGCTCCCG GCATCCGCTT ACAGACAAGC TGTGACCGTC TCCGGGAGCT GCATGTGTCA GAGGTTTTCA CCGTCATCAC CGAAACGCGC

11801 GAGGCAGCTG CGGTAAAGCT CATCAGCGTG GTCGTGAAGC GATTCACAGA TGTCTGCCTG TTCATCCGCG TCCAGCTCGT TGAGTTTCTC CAGAAGCGTT

11901 AATGTCTGGC TTCTGATAAA GCGGGCCATG TTAAGGGCGG TTTTTTCCTG TTTGGTCACT GATGCCTCCG TGTAAGGGGG ATTTCTGTTC ATGGGGGTAA

12001 TGATACCGAT GAAACGAGAG AGGATGCTCA CGATACGGGT TACTGATGAT GAACATGCCC GGTTACTGGA ACGTTGTGAG GGTAAACAAC TGGCGGTATG

12101 GATGCGGCGG GACCAGAGAA AAATCACTCA GGGTCAATGC CAGCGCTTCG TTAATACAGA TGTAGGTGTT CCACAGGGTA GCCAGCAGCA TCCTGCGATG

12201 CAGATCCGGA ACATAATGGT GCAGGGCGCT GACTTCCGCG TTTCCAGACT TTACGAAACA CGGAAACCGA AGACCATTCA TGTTGTTGCT CAGGTCGCAG

12301 ACGTTTTGCA GCAGCAGTCG CTTCACGTTC GCTCGCGTAT CGGTGATTCA TTCTGCTAAC CAGTAAGGCA ACCCCGCCAG CCTAGCCGGG TCCTCAACGA

12401 CAGGAGCACG ATCATGCGCA CCCGTGGCCA GGACCCAACG CTGCCCGAGA TGCGCCGCGT GCGGCTGCTG GAGATGGCGG ACGCGATGGA TATGTTCTGC

12501 CAAGGGTTGG TTTGCGCATT CACAGTTCTC CGCAAGAATT GATTGGCTCC AATTCTTGGA GTGGTGAATC CGTTAGCGAG GTGCCGCCGG CTTCCATTCA

12601 GGTCGAGGTG GCCCGGCTCC ATGCACCGCG ACGCAACGCG GGGAGGCAGA CAAGGTATAG GGCGGCGCCT ACAATCCATG CCAACCCGTT CCATGTGCTC

12701 GCCGAGGCGG CATAAATCGC CGTGACGATC AGCGGTCCAA TGATCGAAGT TAGGCTGGTA AGAGCCGCGA GCGATCCTTG AAGCTGTCCC TGATGGTCGT

12801 CATCTACCTG CCTGGACAGC ATGGCCTGCA ACGCGGGCAT CCCGATGCCG CCGGAAGCGA GAAGAATCAT AATGGGGAAG GCCATCCAGC CTCGCGTCGC

12901 GAACGCCAGC AAGACGTAGC CCAGCGCGTC GGCCGCCATG CCGGCGATAA TGGCCTGCTT CTCGCCGAAA CGTTTGGTGG CGGGACCAGT GACGAAGGCT

13001 TGAGCGAGGG CGTGCAAGAT TCCGAATACC GCAAGCGACA GGCCGATCAT CGTCGCGCTC CAGCGAAAGC GGTCCTCGCC GAAAATGACC CAGAGCGCTG

13101 CCGGCACCTG TCCTACGAGT TGCATGATAA AGAAGACAGT CATAAGTGCG GCGACGATAG TCATGCCCCG CGCCCACCGG AAGGAGCTGA CTGGGTTGAA

13201 GGCTCTCAAG GGCATCGGTC GACGCTCTCC CTTATGCGAC TCCTGCATTA GGAAGCAGCC CAGTAGTAGG TTGAGGCCGT TGAGCACCGC CGCCGCAAGG

13301 AATGGTGCAT GCAAGGAGAT GGCGCCCAAC AGTCCCCCGG CCACGGGGCC TGCCACCATA CCCACGCCGA AACAAGCGCT CATGAGCCCG AAGTGGCGAG

13401 CCCGATCTTC CCCATCGGTG ATGTCGGCGA TATAGGCGCC AGCAACCGCA CCTGTGGCGC CGGTGATGCC GGCCACGATG CGTCCGGCGT AGAG
