# Supplementary material for: Development of a Transformation System for Chlamydia trachomatis: Restoration of Glycogen Biosynthesis by Acquisition of a Plasmid Shuttle Vector
Source: PLoS Pathog. 2011 Sep 22;7(9):e1002258. doi: 10.1371/journal.ppat.1002258 (PMC3178582; doi:10.1371/journal.ppat.1002258)
Supplement: Figure S5 — Plasmid pRSGFPCAT features and sequence. The plasmid pRSGFPCAT is a small in house vector (2670 bp) containing the pUC origin and the RSGFP-CAT gene-fusion under the control of the meningococcal class I protein promoter (MCIP, designated nmP in Figure 5) from MC50. It was constructed by multiple cloning steps. Individual fragments were generated by PCR (see the table below for the sources of each fragment). (DOC) [file ppat.1002258.s005.doc]

**Plasmid pRSGFPCAT features and Sequence**

|  |
| --- |

| **Position on pRSGFPCAT** | **Feature** | **Length** | **Source (PCR template)** | **Sequence changes** |
| --- | --- | --- | --- | --- |
| 6🡪665 | *cat* (chloramphenicol acetyl transferase gene) | 660 bp | pBR325 | nt59 T🡪C |
| 735-1408 | pUC ori | 674 bp | pUC18 | nt908 C🡪T |
| 1433🡪1925 | MCIP (meningococcal class 1 protein promoter from MC50, designated nmP in Fig. 5)  (= promoter for PorA, the outer membrane protein from MC50)  *Refer to the paper below. | 493 bp | MCIP in Neisseria meningitidis MC50  The complete MCIP sequence from MC50 is not available in the database, but GenBank X12899.1 contains part of the promoter sequence).  Therefore we used the MCIP sequence from Neisseria meningitidis MC58 for editing (NC_003112.2, nt1468248-1468739 rc). |  |
| 1947🡪2670🡪5 | RSGFP (red-shifted green fluorescent protein gene) | 729 bp | pS65T-C1 from Clontech | nt1950 G🡪A  nt1963 A🡪C  nt2136 T🡪C |

***Barlow AK, Heckels JE, Clarke IN** (1989) The class 1 outer membrane protein of Neisseria meningitidis: gene sequence and structural and immunological similarities to gonococcal porins. **Mol Microbiol. 3**:131-9

**The sequence of pRSGFPCAT (sequence verified)**

1 GATCTATGGA GAAAAAAATC ACTGGATATA CCACCGTTGA TATATCCCAA TGGCATCGCA AAGAACATTT TGAGGCATTT CAGTCAGTTG CTCAATGTAC

101 CTATAACCAG ACCGTTCAGC TGGATATTAC GGCCTTTTTA AAGACCGTAA AGAAAAATAA GCACAAGTTT TATCCGGCCT TTATTCACAT TCTTGCCCGC

201 CTGATGAATG CTCATCCGGA ATTCCGTATG GCAATGAAAG ACGGTGAGCT GGTGATATGG GATAGTGTTC ACCCTTGTTA CACCGTTTTC CATGAGCAAA

301 CTGAAACGTT TTCATCGCTC TGGAGTGAAT ACCACGACGA TTTCCGGCAG TTTCTACACA TATATTCGCA AGATGTGGCG TGTTACGGTG AAAACCTGGC

401 CTATTTCCCT AAAGGGTTTA TTGAGAATAT GTTTTTCGTC TCAGCCAATC CCTGGGTGAG TTTCACCAGT TTTGATTTAA ACGTGGCCAA TATGGACAAC

501 TTCTTCGCCC CCGTTTTCAC CATGGGCAAA TATTATACGC AAGGCGACAA GGTGCTGATG CCGCTGGCGA TTCAGGTTCA TCATGCCGTT TGTGATGGCT

601 TCCATGTCGG CAGAATGCTT AATGAATTAC AACAGTACTG CGATGAGTGG CAGGGCGGGG CGTAAAGATC TCGAGCTCGA TATCTAGATT AATGTCGACA

701 TATGGGTGAG TTTTCGTTCC ACTGAGCGTC AGACCCCGTA GAAAAGATCA AAGGATCTTC TTGAGATCCT TTTTTTCTGC GCGTAATCTG CTGCTTGCAA

801 ACAAAAAAAC CACCGCTACC AGCGGTGGTT TGTTTGCCGG ATCAAGAGCT ACCAACTCTT TTTCCGAAGG TAACTGGCTT CAGCAGAGCG CAGATACCAA

901 ATACTGTTCT TCTAGTGTAG CCGTAGTTAG GCCACCACTT CAAGAACTCT GTAGCACCGC CTACATACCT CGCTCTGCTA ATCCTGTTAC CAGTGGCTGC

1001 TGCCAGTGGC GATAAGTCGT GTCTTACCGG GTTGGACTCA AGACGATAGT TACCGGATAA GGCGCAGCGG TCGGGCTGAA CGGGGGGTTC GTGCACACAG

1101 CCCAGCTTGG AGCGAACGAC CTACACCGAA CTGAGATACC TACAGCGTGA GCTATGAGAA AGCGCCACGC TTCCCGAAGG GAGAAAGGCG GACAGGTATC

1201 CGGTAAGCGG CAGGGTCGGA ACAGGAGAGC GCACGAGGGA GCTTCCAGGG GGAAACGCCT GGTATCTTTA TAGTCCTGTC GGGTTTCGCC ACCTCTGACT

1301 TGAGCGTCGA TTTTTGTGAT GCTCGTCAGG GGGGCGGAGC CTATGGAAAA ACGCCAGCAA CGCGGCCTTT TTACGGTTCC TGGCCTTTTG CTGGCCTTTT

1401 GCTCACATGT TCTTAAACAA GGATCCTTCC CGGCCTGCGC GGGAATGACG GCTGCAGATG CCCGACGGTC TTTATAGCGG ATTAACAAAA ATCAGGACAA

1501 GGCGGCGAAG CCGAAGACAG TACAAATAGC ACGGAACCGA TTCACTTGGT GCTTCAGCAC CTTAGAGAAT CGTTCTCTTT GAGCTAAGGC GAGGCAACGC

1601 CGTACTTGTT TTTGTTAATC CACTATAAAG TGCCGCGTGT GTTTTTTTAT GGCGTTTTAA AAAGCCGAGA CTGCATCCGG GCAGCAGCGC ATCGGCCCGC

1701 ACGAGGTCTG CGCTTGAATT GTGTTGTAGA AACACAACGT TTTTTGAAAA AATAAGCTAT TGTTTTATAT CAAAATATAA TCATTTTTAA AATAAAGGTT

1801 GCGGCATTTA TCAGATATTT GTTCTGAAAA ATGGTTTTTT GCGGGGGGGG GGGTATAATT GAAGACGTAT CGGGTGTTTG CCCGATGTTT TTAGGTTTTT

1901 ATCAAATTTA CAAAAGGAAG CCGATATGGT GGATCCCCGG GTACCAATGA GTAAAGGAGA AGCACTTTTC ACTGGAGTTG TCCCAATTCT TGTTGAATTA

2001 GATGGTGATG TTAATGGGCA CAAATTTTCT GTCAGTGGAG AGGGTGAAGG TGATGCAACA TACGGAAAAC TTACCCTTAA ATTTATTTGC ACTACTGGAA

2101 AACTACCTGT TCCATGGCCA ACACTTGTCA CTACTCTTAC GTATGGTGTT CAATGCTTTT CAAGATACCC AGATCATATG AAACGGCATG ACTTTTTCAA

2201 GAGTGCCATG CCCGAAGGTT ATGTACAGGA AAGAACTATA TTTTTCAAAG ATGACGGGAA CTACAAGACA CGTGCTGAAG TCAAGTTTGA AGGTGATACC

2301 CTTGTTAATA GAATCGAGTT AAAAGGTATT GATTTTAAAG AAGATGGAAA CATTCTTGGA CACAAATTGG AATACAACTA TAACTCACAC AATGTATACA

2401 TCATGGCAGA CAAACAAAAG AATGGAATCA AAGTTAACTT CAAAATTAGA CACAACATTG AAGATGGAAG CGTTCAACTA GCAGACCATT ATCAACAAAA

2501 TACTCCAATT GGCGATGGCC CTGTCCTTTT ACCAGACAAC CATTACCTGT CCACACAATC TGCCCTTTCG AAAGATCCCA ACGAAAAGAG AGACCACATG

2601 GTCCTTCTTG AGTTTGTAAC AGCTGCTGGG ATTACACATG GCATGGATGA ACTATACAAG TCCGGACTCA
