# Supplementary material for: Development of a Transformation System for Chlamydia trachomatis: Restoration of Glycogen Biosynthesis by Acquisition of a Plasmid Shuttle Vector
Source: PLoS Pathog. 2011 Sep 22;7(9):e1002258. doi: 10.1371/journal.ppat.1002258 (PMC3178582; doi:10.1371/journal.ppat.1002258)
Supplement: Figure S7 — Plasmid pGFP::SW2 features and sequence. The pGFP::SW2 (11539 bp) was created by inserting the C. trachomatis SW2 plasmid (7169 bp, GenBank: FM865439.1) Bam HI fragment into the pSP73 cloning vector (2464 bp, GenBank: X65333.2) Bam HI site (forming an intermediate construct pSP73::SW2), then inserting the Pst I/Sal I fragment (RSGFPCAT cassette) from pRSGFPCAT (2670 bp, to be deposited in GenBank) into Pst I/Sal I sites of pSP73::SW2 (on pSP73 backbone). The promoter for GFPCAT expression is the meningococcal class I protein promoter (MCIP, designated nmP in Figure 5) for outer membrane protein PorA from Neisseria meningitidis MC50. The plasmids pRSGFPCAT and pSW2, and the sequences around cloning sites of pGFP::SW2 were verified. (DOC) [file ppat.1002258.s007.doc]

**Plasmid pGFP::SW2 features and sequence**

| **Position on pGFP::SW2** | **Feature** | **Length** | **Source** | **GenBank #** |
| --- | --- | --- | --- | --- |
| 1*-12 | pSP73 *Sal* I-*Bam* HI fragment | 12 bp | pSP73 |  |
| 13-7181 | pSW2 *Bam* HI fragment | 7169 bp | pSW2 | FM865439.1 |
| 7182-9631 | pSP73 *Bam* HI-*Pst* I fragment | 2450 bp | pSP73 | X65333.2 |
| 9632-11539 | pRSGFPCAT *Pst* I-*Sal* I fragment | 1908 bp | pRSGFPCAT | to be deposited in GenBank |
|  |  |  |  |  |
| 1114🡪122 | CDS2 | 993 bp | pSW2 |  |
|  |  |  |  |  |
| 7698🡪8558 | *bla* | 861 bp | pSP73 |  |
| 8703🡪9376 | pUC ori | 674 bp | pSP73 |  |
|  |  |  |  |  |
| 9632🡪10100 | nmP | 469 bp | pRSGFPCAT |  |
| 10122🡪11510 | RSGFPCAT | 1389 bp | pRSGFPCAT |  |

* Plasmid nucleotide numbering starts at the unique Sal I restriction site.

The sequence of pGFP::SW2 (all the pSW2 sequence and the sequences around the cloning sites have been verified).

1 TCGACTCTAG AGGATCCGTT TGTTCTGGGG AAGAGGTAAT TCCTCTAGTA CAAACACCCA CAATATTGTG ATATAATTAA AATTATATTC ATATTCTGTT

101 GCCAGAAAAA ACACCTTTAG GCTATATTAG AGCCAGCTTC TTTGAAGCGT TGTCTTCTCG AGAAGATTTA TCGTACGCAA ATATCATCTT TGCGGTTGCG

201 TGTCCTGTGA CCTTCATTAT GTCGGAGTCT GAGCACCCTA GGCGTTTGTA CTCCGTCACA GCGGTTGCTC GAAGCACGTG CGGGGTTATT TTAAAAGGGA

301 TTGCAGCTTG TAGTCCTGCT TGAGAGAACG TGCGGGCGAT TTGCCTTAAC CCCACCATTT TTCCGGAGCG AGTTACGAAG ACAAAACCTC TTCGTTGACC

401 GATGTACTCT TGTAGAAAGT GCATAAACTT CTGAGGATAA GTTATAATAA TCCTCTTTTC TGTCTGACGG TTCTTAAGCT GGGAGAAAGA AATGGTAGCT

501 TGTTGGAAAC AAATCTGACT AATCTCCAAG CTTAAGACTT CAGAGGAGCG TTTACCTCCT TGGAGCATTG TCTGGGCGAT CAACCAATCC CGGGCATTGA

601 TTTTTTTTAG CTCTTTTAGG AAGGATGCTG TTTGCAAACT GTTCATCGCA TCCGTTTTTA CTATTTCCCT GGTTTTAAAA AATGTTCGAC TATTTTCTTG

701 TTTAGAAGGT TGCGCTATAG CGACTATTCC TTGAGTCATC CTGTTTAGGA ATCTTGTTAA GGAAATATAG CTTGCTGCTC GAACTTGTTT AGTACCTTCG

801 GTCCAAGAAG TCTTGGCAGA GGAAACTTTT TTAATCGCAT CTAGGATTAG ATTATGATTT AAAAGGGAAA ACTCTTGCAG ATTCATATCC AAGGACAATA

901 GACCAATCTT TTCTAAAGAC AAAAAAGATC CTCGATATGA TCTACAAGTA TGTTTGTTGA GTGATGCGGT CCAATGCATA ATAACTTCGA ATAAGGAGAA

1001 GCTTTTCATG CGTTTCCAAT AGGATTCTTG GCGAATTTTT AAAACTTCCT GATAAGACTT TTCACTATAT TCTAACGACA TTTCTTGCTG CAAAGATAAA

1101 ATCCCTTTAC CCATGAAATC CCTCGTGATA TAACCTATCC GTAAAATGTC CTGATTAGTG AAATAATCAG GTTGTTAACA GGATAGCACG CTCGGTATTT

1201 TTTTATATAA ACAGGTTGTT AACAGGATAG CACGCTCGGT ATTTTTTTAT ATAAACATGA AAACTCGTTC CGAAATAGAA AATCGCATGC AAGATATCGA

1301 GTATGCGTTG TTAGGTAAAG CTCTGATATT TGAAGACTCT ACTGAGTATA TTCTGAGGCA GCTTGCTAAT TATGAGTTTA AGTGTTCTCA TCATAAAAAC

1401 ATATTCATAG TATTTAAATA CTTAAAAGAC AATGGATTAC CTATAACTGT AGACTCGGCT TGGGAAGAGC TTTTGCGGCG TCGTATCAAA GATATGGACA

1501 AATCGTATCT CGGGTTAATG TTGCATGATG CTTTATCAAA TGACAAGCTT AGATCCGTTT CTCATACGGT TTTCCTCGAT GATTTGAGCG TGTGTAGCGC

1601 TGAAGAAAAT TTGAGTAATT TCATTTTCCG CTCGTTTAAT GAGTACAATG AAAATCCATT GCGTAGATCT CCGTTTCTAT TGCTTGAGCG TATAAAGGGA

1701 AGGCTTGATA GTGCTATAGC AAAGACTTTT TCTATTCGCA GCGCTAGAGG CCGGTCTATT TATGATATAT TCTCACAGTC AGAAATTGGA GTGCTGGCTC

1801 GTATAAAAAA AAGACGAGTA GCGTTCTCTG AGAATCAAAA TTCTTTCTTT GATGGCTTCC CAACAGGATA CAAGGATATT GATGATAAAG GAGTTATCTT

1901 AGCTAAAGGT AATTTCGTGA TTATAGCAGC TAGACCATCT ATAGGGAAAA CAGCTTTAGC TATAGACATG GCGATAAATC TTGCGGTTAC TCAACAGCGT

2001 AGAGTTGGTT TCCTATCTCT AGAAATGAGC GCAGGTCAAA TTGTTGAGCG GATTATTGCT AATTTAACAG GAATATCTGG TGAAAAATTA CAAAGAGGGG

2101 ATCTCTCTAA AGAAGAATTA TTCCGAGTAG AAGAAGCTGG AGAAACGGTT AGAGAATCAC ATTTTTATAT CTGCAGTGAT AGTCAGTATA AGCTTAACTT

2201 AATCGCGAAT CAGATCCGGT TGCTGAGAAA AGAAGATCGA GTAGACGTAA TATTTATCGA TTACTTGCAG TTGATCAACT CATCGGTTGG AGAAAATCGT

2301 CAAAATGAAA TAGCAGATAT ATCTAGAACC TTAAGAGGTT TAGCCTCAGA GCTAAACATT CCTATAGTTT GTTTATCCCA ACTATCTAGA AAAGTTGAGG

2401 ATAGAGCAAA TAAAGTTCCC ATGCTTTCAG ATTTGCGAGA CAGCGGTCAA ATAGAGCAAG ACGCAGATGT GATTTTGTTT ATCAATAGGA AGGAATCGTC

2501 TTCTAATTGT GAGATAACTG TTGGGAAAAA TAGACATGGA TCGGTTTTCT CTTCGGTATT ACATTTCGAT CCAAAAATTA GTAAATTCTC CGCTATTAAA

2601 AAAGTATGGT AAATTATAGT AACTGCCACT TCATCAAAAG TCCTATCCAC CTTGAAAATC AGAAGTTTGG AAGAAGACCT GGTCAATCTA TTAAGATATC

2701 TCCCAAATTG GCTCAAAATG GGATGGTAGA AGTTATAGGT CTTGATTTTC TTTCATCTCA TTACCATGCA TTAGCAGCTA TCCAAAGATT ACTGACCGCA

2801 ACGAATTACA AGGGGAACAC AAAAGGGGTT GTTTTATCCA GAGAATCAAA TAGTTTTCAA TTTGAAGGAT GGATACCAAG AATCCGTTTT ACAAAAACTG

2901 AATTCTTAGA GGCTTATGGA GTTAAGCGGT ATAAAACATC CAGAAATAAG TATGAGTTTA GTGGAAAAGA AGCTGAAACT GCTTTAGAAG CCTTATACCA

3001 TTTAGGACAT CAACCGTTTT TAATAGTGGC AACTAGAACT CGATGGACTA ATGGAACACA AATAGTAGAC CGTTACCAAA CTCTTTCTCC GATCATTAGG

3101 ATTTACGAAG GATGGGAAGG TTTAACTGAC GAAGAAAATA TAGATATAGA CTTAACACCT TTTAATTCAC CACCTACACG GAAACATAAA GGGTTCGTTG

3201 TAGAGCCATG TCCTATCTTG GTAGATCAAA TAGAATCCTA CTTTGTAATC AAGCCTGCAA ATGTATACCA AGAAATAAAA ATGCGTTTCC CAAATGCATC

3301 AAAGTATGCT TACACATTTA TCGACTGGGT GATTACAGCA GCTGCGAAAA AGAGACGAAA ATTAACTAAG GATAATTCTT GGCCAGAAAA CTTGTTATTA

3401 AACGTTAACG TTAAAAGTCT TGCATATATT TTAAGGATGA ATCGGTACAT CTGTACAAGG AACTGGAAAA AAATCGAGTT AGCTATCGAT AAATGTATAG

3501 AAATCGCCAT TAAGCTTGGC TGGTTATCTA GAAGAAAACG CATTGAATTT CTGGATTCTT CTAAACTCTC TAAAAAAGAA ATTCTATATC TAAATAAAGA

3601 GCGCTTTGAA GAAATAACTA AGAAATCTAA AGAACAAATG GAACAATTAG AACAAGAATC TATTAATTAA TAGCAAGCTT GAAACTAAAA ACCTAATTTA

3701 TTTAAAGCTC AAAATAAAAA AGAGTTTTAA AATGGGAAAT TCTGGTTTTT ATTTGTATAA CACTGAAAAC TGCGTCTTTG CTGATAATAT CAAAGTTGGG

3801 CAAATGACAG AGCCGCTCAA GGACCAGCAA ATAATCCTTG GGACAACATC AACACCTGTC GCAGCCAAAA TGACAGCTTC TGATGGAATA TCTTTAACAG

3901 TCTCCAATAA TTCATCAACC AATGCTTCTA TTACAATTGG TTTGGATGCG GAAAAAGCTT ACCAGCTTAT TCTAGAAAAG TTGGGAGATC AAATTCTTGA

4001 TGGAATTGCT GATACTATTG TTGATAGTAC AGTCCAAGAT ATTTTAGACA AAATCAAAAC AGACCCTTCT CTAGGTTTGT TGAAAGCTTT TAACAACTTT

4101 CCAATCACTA ATAAAATTCA ATGCAACGGG TTATTCACTC CCAGTAACAT TGAAACTTTA TTAGGAGGAA CTGAAATAGG AAAATTCACA GTCACACCCA

4201 AAAGCTCTGG GAGCATGTTC TTAGTCTCAG CAGATATTAT TGCATCAAGA ATGGAAGGCG GCGTTGTTCT AGCTTTGGTA CGAGAAGGTG ATTCTAAGCC

4301 CTGCGCGATT AGTTATGGAT ACTCATCAGG CATTCCTAAT TTATGTAGTC TAAGAACCAG TATTACTAAT ACAGGATTGA CTCCGACAAC GTATTCATTA

4401 CGTGTAGGCG GTTTAGAAAG CGGTGTGGTA TGGGTTAATG CCCTTTCTAA TGGCAATGAT ATTTTAGGAA TAACAAATAC TTCTAATGTA TCTTTTTTAG

4501 AGGTAATACC TCAAACAAAC GCTTAAACAA TTTTTATTGG ATTTTTCTTA TAGGTTTTAT ATTTAGAGAA AACAGTTCGA ATTACGGGGT TTGTTATGCA

4601 AAATAAAAGA AAAGTGAGGG ACGATTTTAT TAAAATTGTT AAAGATGTGA AAAAAGATTT CCCCGAATTA GACCTAAAAA TACGAGTAAA CAAGGAAAAA

4701 GTAACTTTCT TAAATTCTCC CTTAGAACTC TACCATAAAA GTGTCTCACT AATTCTAGGA CTGCTTCAAC AAATAGAAAA CTCTTTAGGA TTATTCCCAG

4801 ACTCTCCTGT TCTTGAAAAA TTAGAGGATA ACAGTTTAAA GCTAAAAAAG GCTTTGATTA TGCTTATCTT GTCTAGAAAA GACATGTTTT CCAAGGCTGA

4901 ATAGACAACT TACTCTAACG TTGGAGTTGA TTTGCACACC TTAGTTTTTT GCTCTTTTAA GGGAGGAACT GGAAAAACAA CACTTTCTCT AAACGTGGGA

5001 TGCAACTTGG CCCAATTTTT AGGGAAAAAA GTGTTACTTG CTGACCTAGA CCCGCAATCC AATTTATCTT CTGGATTGGG GGCTAGTGTC AGAAGTGACC

5101 AAAAAGGCTT GCACGACATA GTATACACAT CAAACGATTT AAAATCAATC ATTTGCGAAA CAAAAAAAGA TAGTGTGGAC CTAATTCCTG CATCATTTTC

5201 ATCCGAACAG TTTAGAGAAT TGGATATTCA TAGAGGACCT AGTAACAACT TAAAGTTATT TCTGAATGAG TACTGCGCTC CTTTTTATGA CATCTGCATA

5301 ATAGACACTC CACCTAGCCT AGGAGGGTTA ACGAAAGAAG CTTTTGTTGC AGGAGACAAA TTAATTGCTT GTTTAACTCC AGAACCTTTT TCTATTCTAG

5401 GGTTACAAAA GATACGTGAA TTCTTAAGTT CGGTCGGAAA ACCTGAAGAA GAACACATTC TTGGAATAGC TTTGTCTTTT TGGGATGATC GTAACTCGAC

5501 TAACCAAATG TATATAGACA TTATCGAGTC TATTTACAAA AACAAGCTTT TTTCAACAAA AATTCGTCGA GATATTTCTC TCAGCCGTTC TCTTCTTAAA

5601 GAAGATTCTG TAGCTAATGT CTATCCAAAT TCTAGGGCCG CAGAAGATAT TCTGAAGTTA ACGCATGAAA TAGCAAATAT TTTGCATATC GAATATGAAC

5701 GAGATTACTC TCAGAGGACA ACGTGAACAA ACTAAAAAAA GAAGCGGATG TCTTTTTTAA AAAAAATCAA ACTGCCGCTT CTCTAGATTT TAAGAAGACG

5801 CTTCCCTCCA TTGAACTATT CTCAGCAACT TTGAATTCTG AGGAAAGTCA GAGTTTGGAT CGATTATTTT TATCAGAGTC CCAAAACTAT TCGGATGAAG

5901 AATTTTATCA AGAAGACATC CTAGCGGTAA AACTGCTTAC TGGTCAGATA AAATCCATAC AGAAGCAACA CGTACTTCTT TTAGGAGAAA AAATCTATAA

6001 TGCTAGAAAA ATCCTGAGTA AGGATCACTT CTCCTCAACA ACTTTTTCAT CTTGGATAGA GTTAGTTTTT AGAACTAAGT CTTCTGCTTA CAATGCTCTT

6101 GCATATTACG AGCTTTTTAT AAACCTCCCC AACCAAACTC TACAAAAAGA GTTTCAATCG ATCCCCTATA AATCCGCATA TATTTTGGCC GCTAGAAAAG

6201 GCGATTTAAA AACCAAGGTC GATGTGATAG GGAAAGTATG TGGAATGTCG AACTCATCGG CGATAAGGGT GTTGGATCAA TTTCTTCCTT CATCTAGAAA

6301 CAAAGACGTT AGAGAAACGA TAGATAAGTC TGATTCAGAG AAGAATCGCC AATTATCTGA TTTCTTAATA GAGATACTTC GCATCATGTG TTCCGGAGTT

6401 TCTTTGTCCT CCTATAACGA AAATCTTCTA CAACAGCTTT TTGAACTTTT TAAGCAAAAG AGCTGATCCT CCGTCAGCTC ATATATATAT ATCTATTATA

6501 TATATATATT TAGGGATTTG ATTTCACGAG AGAGATTTGC AACTCTTGGT GGTAGACTTT GCAACTCTTG GTGGTAGACT TTGCAACTCT TGGTGGTAGA

6601 CTTTGCAACT CTTGGTGGTA GACTTGGTCA TAATGGACTT TTGTTAAAAA ATTTCTTAAA ATCTTAGAGC TCCGATTTTG AATAGCTTTG GTTAAGAAAA

6701 TGGGCTCGAT GGCTTTCCAT AAAAGTAGAT TGTTTTTAAC TTTTGGGGAC GCGTCGGAAA TTTGGTTATC TACTTTATCT TATCTAACTA GAAAAAATTA

6801 TGCGTCTGGG ATTAACTTTC TTGTTTCTTT AGAGATTCTG GATTTATCGG AAACCTTGAT AAAGGCTATT TCTCTTGACC ACAGCGAATC TTTGTTTAAA

6901 ATCAAGTCTC TAGATGTTTT TAATGGAAAA GTTGTTTCAG AGGCATCTAA ACAGGCTAGA GCGGCATGCT ACATATCTTT CACAAAGTTT TTGTATAGAT

7001 TGACCAAGGG ATATATTAAA CCCGCTATTC CATTGAAAGA TTTTGGAAAC ACTACATTTT TTAAAATCCG AGACAAAATC AAAACAGAAT CGATTTCTAA

7101 GCAGGAATGG ACAGTTTTTT TTGAAGCGCT CCGGATAGTG AATTATAGAG ACTATTTAAT CGGTAAATTG ATTGTACAAG GGATCCCCGG GTACCGAGCT

7201 CGAATTCATC GATGATATCA GATCTGGTTC TATAGTGTCA CCTAAATCGT ATGTGTATGA TACATAAGGT TATGTATTAA TTGTAGCCGC GTTCTAACGA

7301 CAATATGTCC ATATGGTGCA CTCTCAGTAC AATCTGCTCT GATGCCGCAT AGTTAAGCCA GCCCCGACAC CCGCCAACAC CCGCTGACGC GCCCTGACGG

7401 GCTTGTCTGC TCCCGGCATC CGCTTACAGA CAAGCTGTGA CCGTCTCCGG GAGCTGCATG TGTCAGAGGT TTTCACCGTC ATCACCGAAA CGCGCGAGAC

7501 GAAAGGGCCT CGTGATACGC CTATTTTTAT AGGTTAATGT CATGATAATA ATGGTTTCTT AGACGTCAGG TGGCACTTTT CGGGGAAATG TGCGCGGAAC

7601 CCCTATTTGT TTATTTTTCT AAATACATTC AAATATGTAT CCGCTCATGA GACAATAACC CTGATAAATG CTTCAATAAT ATTGAAAAAG GAAGAGTATG

7701 AGTATTCAAC ATTTCCGTGT CGCCCTTATT CCCTTTTTTG CGGCATTTTG CCTTCCTGTT TTTGCTCACC CAGAAACGCT GGTGAAAGTA AAAGATGCTG

7801 AAGATCAGTT GGGTGCACGA GTGGGTTACA TCGAACTGGA TCTCAACAGC GGTAAGATCC TTGAGAGTTT TCGCCCCGAA GAACGTTTTC CAATGATGAG

7901 CACTTTTAAA GTTCTGCTAT GTGGCGCGGT ATTATCCCGT ATTGACGCCG GGCAAGAGCA ACTCGGTCGC CGCATACACT ATTCTCAGAA TGACTTGGTT

8001 GAGTACTCAC CAGTCACAGA AAAGCATCTT ACGGATGGCA TGACAGTAAG AGAATTATGC AGTGCTGCCA TAACCATGAG TGATAACACT GCGGCCAACT

8101 TACTTCTGAC AACGATCGGA GGACCGAAGG AGCTAACCGC TTTTTTGCAC AACATGGGGG ATCATGTAAC TCGCCTTGAT CGTTGGGAAC CGGAGCTGAA

8201 TGAAGCCATA CCAAACGACG AGCGTGACAC CACGATGCCT GTAGCAATGG CAACAACGTT GCGCAAACTA TTAACTGGCG AACTACTTAC TCTAGCTTCC

8301 CGGCAACAAT TAATAGACTG GATGGAGGCG GATAAAGTTG CAGGACCACT TCTGCGCTCG GCCCTTCCGG CTGGCTGGTT TATTGCTGAT AAATCTGGAG

8401 CCGGTGAGCG TGGGTCTCGC GGTATCATTG CAGCACTGGG GCCAGATGGT AAGCCCTCCC GTATCGTAGT TATCTACACG ACGGGGAGTC AGGCAACTAT

8501 GGATGAACGA AATAGACAGA TCGCTGAGAT AGGTGCCTCA CTGATTAAGC ATTGGTAACT GTCAGACCAA GTTTACTCAT ATATACTTTA GATTGATTTA

8601 AAACTTCATT TTTAATTTAA AAGGATCTAG GTGAAGATCC TTTTTGATAA TCTCATGACC AAAATCCCTT AACGTGAGTT TTCGTTCCAC TGAGCGTCAG

8701 ACCCCGTAGA AAAGATCAAA GGATCTTCTT GAGATCCTTT TTTTCTGCGC GTAATCTGCT GCTTGCAAAC AAAAAAACCA CCGCTACCAG CGGTGGTTTG

8801 TTTGCCGGAT CAAGAGCTAC CAACTCTTTT TCCGAAGGTA ACTGGCTTCA GCAGAGCGCA GATACCAAAT ACTGTCCTTC TAGTGTAGCC GTAGTTAGGC

8901 CACCACTTCA AGAACTCTGT AGCACCGCCT ACATACCTCG CTCTGCTAAT CCTGTTACCA GTGGCTGCTG CCAGTGGCGA TAAGTCGTGT CTTACCGGGT

9001 TGGACTCAAG ACGATAGTTA CCGGATAAGG CGCAGCGGTC GGGCTGAACG GGGGGTTCGT GCACACAGCC CAGCTTGGAG CGAACGACCT ACACCGAACT

9101 GAGATACCTA CAGCGTGAGC ATTGAGAAAG CGCCACGCTT CCCGAAGGGA GAAAGGCGGA CAGGTATCCG GTAAGCGGCA GGGTCGGAAC AGGAGAGCGC

9201 ACGAGGGAGC TTCCAGGGGG AAACGCCTGG TATCTTTATA GTCCTGTCGG GTTTCGCCAC CTCTGACTTG AGCGTCGATT TTTGTGATGC TCGTCAGGGG

9301 GGCGGAGCCT ATGGAAAAAC GCCAGCAACG CGGCCTTTTT ACGGTTCCTG GCCTTTTGCT GGCCTTTTGC TCACATGTTC TTTCCTGCGT TATCCCCTGA

9401 TTCTGTGGAT AACCGTATTA CCGCCTTTGA GTGAGCTGAT ACCGCTCGCC GCAGCCGAAC GACCGAGCGC AGCGAGTCAG TGAGCGAGGA AGCGGAAGAG

9501 CGCCCAATAC GCAAACCGCC TCTCCCCGCG CGTTGGCCGA TTCATTAATG CAGGTTAACC TGGCTTATCG AAATTAATAC GACTCACTAT AGGGAGACCG

9601 GCCTCGAGCA GCTGAAGCTT GCATGCCTGC AGATGCCCGA CGGTCTTTAT AGCGGATTAA CAAAAATCAG GACAAGGCGG CGAAGCCGAA GACAGTACAA

9701 ATAGCACGGA ACCGATTCAC TTGGTGCTTC AGCACCTTAG AGAATCGTTC TCTTTGAGCT AAGGCGAGGC AACGCCGTAC TTGTTTTTGT TAATCCACTA

9801 TAAAGTGCCG CGTGTGTTTT TTTATGGCGT TTTAAAAAGC CGAGACTGCA TCCGGGCAGC AGCGCATCGG CCCGCACGAG GTCTGCGCTT GAATTGTGTT

9901 GTAGAAACAC AACGTTTTTT GAAAAAATAA GCTATTGTTT TATATCAAAA TATAATCATT TTTAAAATAA AGGTTGCGGC ATTTATCAGA TATTTGTTCT

10001 GAAAAATGGT TTTTTGCGGG GGGGGGGGTA TAATTGAAGA CGTATCGGGT GTTTGCCCGA TGTTTTTAGG TTTTTATCAA ATTTACAAAA GGAAGCCGAT

10101 ATGGTGGATC CCCGGGTACC AATGAGTAAA GGAGAAGCAC TTTTCACTGG AGTTGTCCCA ATTCTTGTTG AATTAGATGG TGATGTTAAT GGGCACAAAT

10201 TTTCTGTCAG TGGAGAGGGT GAAGGTGATG CAACATACGG AAAACTTACC CTTAAATTTA TTTGCACTAC TGGAAAACTA CCTGTTCCAT GGCCAACACT

10301 TGTCACTACT CTTACGTATG GTGTTCAATG CTTTTCAAGA TACCCAGATC ATATGAAACG GCATGACTTT TTCAAGAGTG CCATGCCCGA AGGTTATGTA

10401 CAGGAAAGAA CTATATTTTT CAAAGATGAC GGGAACTACA AGACACGTGC TGAAGTCAAG TTTGAAGGTG ATACCCTTGT TAATAGAATC GAGTTAAAAG

10501 GTATTGATTT TAAAGAAGAT GGAAACATTC TTGGACACAA ATTGGAATAC AACTATAACT CACACAATGT ATACATCATG GCAGACAAAC AAAAGAATGG

10601 AATCAAAGTT AACTTCAAAA TTAGACACAA CATTGAAGAT GGAAGCGTTC AACTAGCAGA CCATTATCAA CAAAATACTC CAATTGGCGA TGGCCCTGTC

10701 CTTTTACCAG ACAACCATTA CCTGTCCACA CAATCTGCCC TTTCGAAAGA TCCCAACGAA AAGAGAGACC ACATGGTCCT TCTTGAGTTT GTAACAGCTG

10801 CTGGGATTAC ACATGGCATG GATGAACTAT ACAAGTCCGG ACTCAGATCT ATGGAGAAAA AAATCACTGG ATATACCACC GTTGATATAT CCCAATGGCA

10901 TCGCAAAGAA CATTTTGAGG CATTTCAGTC AGTTGCTCAA TGTACCTATA ACCAGACCGT TCAGCTGGAT ATTACGGCCT TTTTAAAGAC CGTAAAGAAA

11001 AATAAGCACA AGTTTTATCC GGCCTTTATT CACATTCTTG CCCGCCTGAT GAATGCTCAT CCGGAATTCC GTATGGCAAT GAAAGACGGT GAGCTGGTGA

11101 TATGGGATAG TGTTCACCCT TGTTACACCG TTTTCCATGA GCAAACTGAA ACGTTTTCAT CGCTCTGGAG TGAATACCAC GACGATTTCC GGCAGTTTCT

11201 ACACATATAT TCGCAAGATG TGGCGTGTTA CGGTGAAAAC CTGGCCTATT TCCCTAAAGG GTTTATTGAG AATATGTTTT TCGTCTCAGC CAATCCCTGG

11301 GTGAGTTTCA CCAGTTTTGA TTTAAACGTG GCCAATATGG ACAACTTCTT CGCCCCCGTT TTCACCATGG GCAAATATTA TACGCAAGGC GACAAGGTGC

11401 TGATGCCGCT GGCGATTCAG GTTCATCATG CCGTTTGTGA TGGCTTCCAT GTCGGCAGAA TGCTTAATGA ATTACAACAG TACTGCGATG AGTGGCAGGG

11501 CGGGGCGTAA AGATCTCGAG CTCGATATCT AGATTAATG
