# Supplementary material for: Development of a Transformation System for Chlamydia trachomatis: Restoration of Glycogen Biosynthesis by Acquisition of a Plasmid Shuttle Vector
Source: PLoS Pathog. 2011 Sep 22;7(9):e1002258. doi: 10.1371/journal.ppat.1002258 (PMC3178582; doi:10.1371/journal.ppat.1002258)
Supplement: Table S1 — Summary of conditions to select penicillin-resistant C. trachomatis transformants (DOC) [file ppat.1002258.s009.doc]

**Summary of conditions used to select penicillin-resistant *C. trachomatis* L2 transformants**

| **Passage** | **Culture vessel** | **Penicillin selection** | **Duration** | **Comments** |
| --- | --- | --- | --- | --- |
| Passage 1  (4 ml of T_0_ is used as the inoculum) | T_75_ flask | 10 units/ml | 2 days | *T_0_ is used to infect McCoy cells in a T_75_ flask, transformants are selected with 10 units/ml of penicillin, applied from the time of infection. Most inclusions are large and vacuolar as previously described (Skilton *et al*, 2009). *Chlamydia* are grown for two days before harvesting as ‘T_1_’ and this is used to infect cells as passage 2. |
| Passage 2  (use T_1_ ) | T_25_ flask | 10 units/ml | 4-7 days | Use half of T_1_ as an inoculum to infect McCoy cells in a T_25_ flask and select with 10 units/ml of penicillin. Some normal inclusions may be observed in this passage. This culture is harvested as T_2_ and passaged again. |
| Passage 3  (use T_2_ ) | T_25_ flask | 10 units/ml | 2-5 days | Use T_2_ as an inoculum to infect McCoy cells in a T_25_ flask and select with 10 units/ml of penicillin. Normal inclusions are routinely recovered in this passage. Harvest this culture is T_3_ and passage. |
| Passage 4  (use T_3_ ) | T_25_ flask | 10 units/ml | 2-5 days | Use T_3_ as an inoculum to infect McCoy cells in a T_25_ flask and select with 10 units/ml of penicillin to amplify transformants, higher concentrations of penicillin (up to 100 units/ml) can be used. |

* T_0_ is the inoculum harvested from the primary transformation mix (*C.trachomatis* EBs / plasmid DNA / McCoy cells) propagated in a single well of a 6-well tray.
